# Bispecific Antibodies for Multiple Myeloma: A Review of Targets, Drugs, Clinical Trials, and Future Directions

**DOI:** 10.3389/fimmu.2020.00501

**Published:** 2020-04-24

**Authors:** Chiara Caraccio, Sachi Krishna, Darci J. Phillips, Christian M. Schürch

**Affiliations:** Baxter Laboratory for Stem Cell Biology, Department of Microbiology and Immunology, Stanford University School of Medicine, Stanford, CA, United States

**Keywords:** BCMA, bispecific antibodies, CD38, clinical trials, high-risk disease, multiple myeloma, review

## Abstract

Multiple myeloma (MM) is a plasma cell malignancy and the second most common hematological neoplasm in adults, comprising 1.8% of all cancers. With an annual incidence of ~30,770 cases in the United States, MM has a high mortality rate, leading to 12,770 deaths per year. MM is a genetically complex, highly heterogeneous malignancy, with significant inter- and intra-patient clonal variability. Recent years have witnessed dramatic improvements in the diagnostics, classification, and treatment of MM. However, patients with high-risk disease have not yet benefited from therapeutic advances. High-risk patients are often primary refractory to treatment or relapse early, ultimately resulting in progression toward aggressive end-stage MM, with associated extramedullary disease or plasma cell leukemia. Therefore, novel treatment modalities are needed to improve the outcomes of these patients. Bispecific antibodies (BsAbs) are immunotherapeutics that simultaneously target and thereby redirect effector immune cells to tumor cells. BsAbs have shown high efficacy in B cell malignancies, including refractory/relapsed acute lymphoblastic leukemia. Various BsAbs targeting MM-specific antigens such as B cell maturation antigen (BCMA), CD38, and CD138 are currently in pre-clinical and clinical development, with promising results. In this review, we outline these advances, focusing on BsAb drugs, their targets, and their potential to improve survival, especially for high-risk MM patients. In combination with current treatment strategies, BsAbs may pave the way toward a cure for MM.

## Introduction

Multiple myeloma (MM) is the second most common hematologic malignancy in adults (1). In the United States in 2018, ~30,770 patients were diagnosed with MM and 12,770 died from their disease, representing 2.9% of all cancer deaths (2). MM is characterized by a clonal expansion of malignantly transformed plasma cells in the bone marrow (BM). These cells produce an excess of monoclonal immunoglobulins, which are secreted into the blood and urine. Major complications in MM patients include tumor-induced bone lesions and associated pathological fractures, anemia, renal failure, and immunodeficiency, leading to impaired quality of life and decreased overall survival (3, 4).

Over the last few decades, novel drug classes such as immunomodulators (e.g., lenalidomide), proteasome inhibitors (e.g., bortezomib), histone deacetylase inhibitors (e.g., panobinostat), and monoclonal antibodies (mAbs) (e.g., daratumumab [anti-CD38]) have significantly improved the response rates and overall survival for MM patients (5, 6). Currently, the median overall survival for MM patients is 5 years (7). However, stratification by disease risk, according to the Revised International Staging System (R-ISS), reveals significant variability: 82% of low-risk, stage I patients survive 5 years, compared to only 40% of high-risk, stage III patients (7). While high-risk MM patients only account for 15 to 20% of newly diagnosed cases, these patients are often primary refractory to treatment or relapse early (8). Additionally, the majority of low-risk MM patients ultimately develop drug-resistant clones, become refractory to treatment and transition to high-risk disease (8–10). These findings underscore the need to identify MM patients who have active high-risk disease, as well as those who are likely to progress, and develop novel treatment strategies targeted at this population.

Bispecific antibodies (BsAbs) offer a promising immunotherapeutic approach for numerous malignancies including MM. Immune effector cell redirecting BsAbs commonly bind to a tumor cell antigen and CD3 on a T cell, resulting in T cell binding to the tumor cell, activation, and tumor cell lysis (11, 12). Since BsAbs directly stimulate CD3 and thus bypass the T cell receptor, they activate T cells independently from antigen presentation on major histocompatibility complex (MHC) class I. In addition, they have the ability to activate T cells in the absence of co-stimulation, bypassing the normal dependence on antigen presenting cells or cytokines and reducing the risk of anergy that accompanies TCR stimulation in the absence of a costimulatory signal (12–20).

Here, we review the potential of BsAbs in MM, with an emphasis on high-risk patients, although the benefits of BsAbs can extend to all MM patients. A brief introduction into MM is followed by an overview of current BsAb strategies. Next, novel BsAb developments and clinical trials for different MM targets are discussed. Finally, the future direction of BsAbs as a MM treatment modality is addressed, along with obstacles that need to be overcome.

## Origins of MM and Features of High-Risk Disease

**Figure 1:**
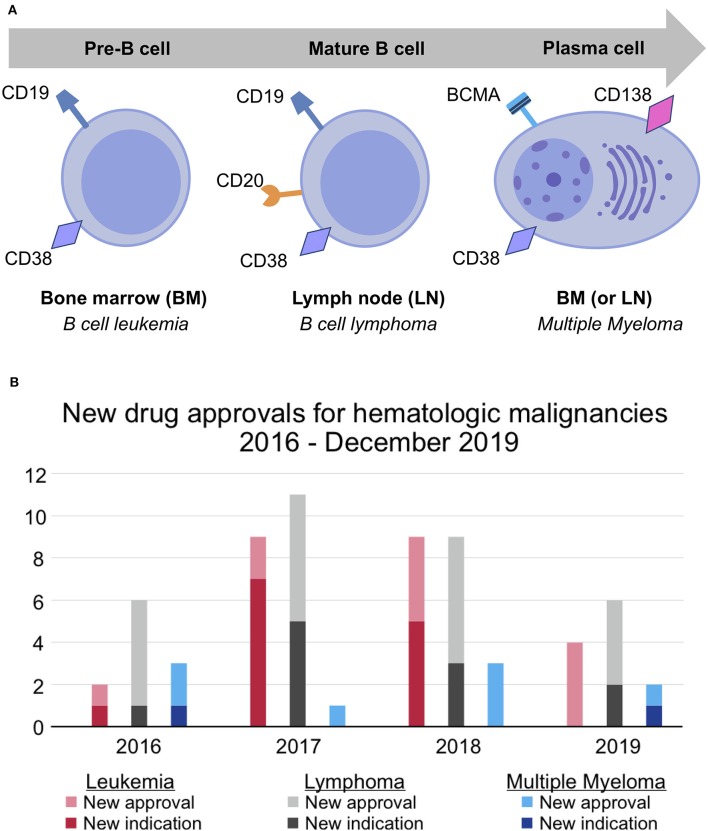
Differences in molecular architecture and therapeutic success for hematologic malignancy subtypes. **(A)** B cell leukemia, B cell lymphoma, and multiple myeloma occur at different stages of the B cell lifecycle. Unlike pre- and mature B-cells, which express CD19 and CD20, plasma cells uniquely express BCMA and CD138. CD38 is expressed at all stages of the B cell lifecycle but is more highly expressed on malignant plasma cells. **(B)** New drug approvals for leukemia, lymphoma, and multiple myeloma between 2016 and December 2019 (21, 22). The pace of drug development for multiple myeloma has failed to keep pace with that of leukemia and lymphoma.

MM is a cancer of plasma cells, which are terminally differentiated B cells. Numerous hematologic malignancies result from the malignant transformation of B cells at different stages in their lifecycle. For instance, B cell leukemias usually arise from BM-residing pre-B cells; B cell lymphomas, from mature B cells that have migrated to lymph nodes; and MM, from long-lived, BM-residing plasma cells (Figure 1A). Malignant B cells and malignant plasma cells have key differences in their molecular architecture. Unlike malignant B cells, malignant plasma cells generally do not express the widely targeted cell-surface proteins CD19 and CD20, although up to 20% of patients have CD20^+^ MM clones (23). As such, most MM patients cannot be treated with many of the newly-approved targeted therapies for B cell leukemias and lymphomas. This is in part reflected by the number of new drug approvals/indications, which was 56 for B cell leukemias/lymphomas and only 10 for MM in the past 4 years [(21, 22); Figure 1B].

MM develops from a pre-malignant condition known as monoclonal gammopathy of undetermined significance (MGUS), which progresses to smoldering MM (SMM) and MM at a rate of 1% per year (24). Virtually all known MM cases are preceded by MGUS, but the vast majority of MGUS cases never develop into MM. MGUS is characterized by low levels of serum M-protein (<3 g/dL) and <10% clonal BM plasma cells, whereas these levels are >3 g/dL and >10% for SMM, respectively (24). Importantly, the proliferation of malignant plasma cells in patients with MGUS and SMM is asymptomatic and these patients do not exhibit end-organ damage. MM patients are further staged into risk categories, which predict prognosis and treatment response. The most commonly used risk stratification model is the R-ISS, but several models exist (Table 1). With ongoing disease progression, MM patients ultimately develop aggressive, end-stage MM in the form of extramedullary disease or plasma cell leukemia. Table 2 details emerging factors associated with high-risk MM.

**Table 1:** MM classification systems and definitions of high-risk disease.

**Classification system**	**Features**	**Model**	**Year introduced**	**References**
Durie-Salmon	Stage III One or more of the following: hemoglobin <8.5 g/dL; serum calcium >12 mg/dL; Advanced lytic bone lesions; high M-component production rates IgG value>7 g/dL, IgA >5 g/dL; urine light chain M-component >12 g/24 h	Tumor Burden/Stage	1975	Durie-Salmon Staging System (25)
International Staging System (ISS)	Stage III Serum β_2_-microglobulin ≥ 5.5 mg/L (other stages consider serum albumin levels as well)	Tumor Burden/ Stage	2005	Greipp et al. (26)
University of Arkansas for Medical Sciences (UAMS)17-gene model	High Risk Deregulated expression of 17 genes (1q32.1, 21q22.3, 1q21.2, 8q23.1, 10q23.31, 12q22, 1p36.21, 3p21.3, 7p14-p13, 1q22, 1q43, 1q31, 1p13.2, 1p22, 1p13.3, 2p22-p21, 6p21)	Cytogenetics	2007	Shaughnessy et al. (27)
Medical Research Council (MRC) Myeloma IX Trial	Adverse lesions defined as +1q21, del(17p13), del(13q14), or adverse IGH translocations t(4;14), t(14;16), or t(14;20) High Risk Presence of >1 adverse lesion Ultra-high Risk Presence of >1 adverse genetic lesions and ISS II or III	Combined Cytogenetics-ISS	2012	Boyd et al. (28)
mSMART	High Risk Genetic abnormalities on t(14;16), t(14;20), del(17p); GEP high risk signature	Cytogenetics	2013	Mikhael et al. (29)
International Myeloma Working Group (IMWG)	High Risk ISS II/III and t(4;14) or del(17p13)	Combined Cytogenetics-ISS	2014	Chng et al. (30)
Revised International Staging System (R-ISS)	Stage III ISS stage III (Serum β_2_-microglobulin 5.5 mg/L) and either: high risk CA by iFISH (presence of del(17p) and/or translocation t(4;14) and/or translocation t(14;16), or high lactate dehydrogenase (LDH) (serum LDH > the upper limit of normal)	Combined Cytogenetics-ISS	2015	Palumbo et al. (7)
mSMART 3.0	High Risk Genetic abnormalities: t(4;14), t(14;16), t(14;20), del(17p), p53 mutation, +1q; RISS stage III; High plasma cell s-phase; high GEP risk signature	Combined Genetics-ISS	2018	Treatment Guidelines (31)

*CA, cytogenetic abnormality; GEP, gene expression profiling; iFISH, interphase fluorescence in situ hybridization; IGH, immunoglobulin heavy; ISS, International Staging System; LDH, lactate dehydrogenase; mSMART, Mayo stratification of myeloma and risk-adapted therapy; R-ISS, Revised International Staging System*.

**Table 2:** Emerging high-risk MM factors.

**Type**	**Factors**	**Year**	**References**
Cytogenetic	t(14;16) (q32;q23); t(14;20) (q32;q23); Del(17p)	2016	Rajkumar (32)
Cytogenetic	FISH: t(4;14), t(14;16), t(14;20), del(17/17p), gain(1q); Non-hyperdiploid karyotype; Karyotype del(13); high-risk GEP70 signature	2016	Sonneveld et al. (33)
Cytogenetic	Primary translocations: t(4;14), t(14;16), t(14;20) Secondary translocations: MYC, jumping translocation 1q Copy change number: Isochromosome formation, hyperhaploidy, gain(1q), del(1p), del(17p) Homozygous inactivation of TSGs: Mutation +/- copy number change Genetic changes associated with DNA repair deficiency: genome-wide loss of heterozygosity	2017	Pawlyn and Morgan (34)
Epigenetic	Epigenetic modifier mutations; histone methylation and acetylation; DNA methylation, measured via mutations in DNA methylation modifiers, e.g., IDH1; microRNA	2017	Pawlyn and Morgan (34)
Bone Lesions	Presence of 3 large focal lesions, with a product of the perpendicular diameters > 5 cm^2^	2018	Rasche et al. (35)

*IDH1, isocitrate dehydrogenase 1; EZH2, enhancer-of-zeste 2 polycomb repressive complex 2 subunit; FISH, fluorescence in situ hybridization; GEP70, 70-gene expression profiling; MM, multiple myeloma; TSG, tumor suppressor gene*.

Genetic and epigenetic events that initiate MM include chromosomal hyperdiploidy, translocations of chromosome 14 (bringing the strong immunoglobulin heavy-chain enhancer into the proximity of oncogenes), the dysregulation of cell cycle genes, abnormalities in signaling pathways, and alteration of DNA methylation (36–38). Further aberrations, including MYC overexpression and mutations in RAS oncogenes, amongst others, are associated with disease progression (34, 36). In contrast to the evolving genetic heterogeneity associated with disease progression, the immune phenotype of MM cells is relatively conserved. For example, the malignant plasma cells in MGUS, SMM, and MM all express the key surface markers B cell maturation antigen (BCMA), CD38, and CD138. While BCMA expression significantly increases with disease progression, changes in CD38 and CD138 levels are less well-characterized (38, 39). In addition to these molecular features, MM cells rely on their BM microenvironment for growth, survival, and the development of therapy-resistant clones. Through cell-cell interactions and the secretion of cytokines, chemokines, and other factors, MM cells proliferate and impair the effector function of neighboring immune cells (38). For instance, in the BM of MM patients, key immunosuppressive cytokines are expressed at high levels (40, 41). These include interleukin-6 (IL-6), which mediates autocrine and paracrine growth of MM cells and inhibits tumor cell apoptosis, as well as TGF-β, which is an immune inhibitory factor that induces IL-6 secretion. Additionally, regulatory T cell (Treg) numbers are increased in MM patients, further suppressing the immune BM milieu (42, 43). The immunosuppressive characteristics of the molecular and cellular constituents of the BM microenvironment aid in disease progression and lead to poor clinical outcomes (37).

Given the immunosuppressive microenvironment of MM, successful therapies must simultaneously destroy malignant plasma cells and restore an effective anti-tumoral immune response (44). Such immunotherapies should (1) target surface molecules that are ideally expressed exclusively or at higher levels in MM cells than normal plasma or other immune cells and (2) bring effector immune cells into contact with MM cells, thereby enhancing effector cell-directed anti-tumor immunity. BsAbs meet these criteria, and therefore represent a next-generation immunotherapy with the potential to provide sustained clinical responses and even a cure for MM patients.

## Bispecific Antibodies: Overview, Designs, and Potential for MM

The idea of using BsAbs to redirect immune cells to tumor cells was first demonstrated in the 1980s and led to several clinical trials (45, 46). Catumaxomab (anti-EpCAM × anti-CD3) was the first BsAb to meet clinical approval by the European Union in 2009 (47). Blinatumomab (anti-CD19 × anti-CD3) was the first BsAb approved by the US Food and Drug Administration (FDA) in 2014 (48–50). Since then, one more BsAb—emicizumab, used to treat hemophilia A—has obtained FDA approval, and there are currently more than 60 BsAbs in various stages of preclinical and clinical development (51, 52). To date, no BsAb has been approved for use in MM patients, although there are 13 currently in clinical trials, and a pilot study evaluating the effects of blinatumomab in relapsed/refractory (R/R) MM patients was initiated in May 2017 (Table 3).

**Table 3:** Clinical trials of BsAbs targeting MM.

**Targets**	**Drug name**	**Design**	**Trial type**	**Estimated enrollment**	**Estimated completion**	**References**
BCMA × CD3	PF-06863135	IgG2a Fc region	Phase 1	80	Early 2022	NCT03269136
						
BCMA × CD3	TNB-383B	IgG4 Fc region	Phase 1	72	Late 2021	NCT03933735
						
BCMA × CD3	REGN5458	Fc region, Fab arms	Phase 1/2	56	Late 2022	NCT03761108
						
BCMA × CD3	REGN5459	Fc region, Fab arms	Phase 1/2	56	Late 2023	NCT04083534
						
BCMA × CD3	CC-93269	Trivalent, Fc region	Phase 1	19	Mid 2022	NCT03486067
						
BCMA × CD3	JNJ-64007957	IgG1 Fc region	Phase 1	120	Mid 2020	NCT03145181
						
BCMA × CD3	AMG420	BiTE	Phase 1	120	Early 2025	NCT02514239
						
BCMA × CD3	AMG701	Half-life extended BiTE (scFvs plus Fc region)	Phase 1	135	Mid 2025	NCT03287908
						
CD38 × CD3	AMG424	Fc region, scFv x Fab arms	Phase 1	20	Late 2022	NCT03445663
						
CD38 × CD3	GBR1342	Fc region, scFv x Fab arms	Phase 1	125	Early 2021	NCT03309111
						
CD19 × CD3	Blinatumomab	BiTE	Phase 1	20	Mid 2020	NCT03173430
						
FcRL5 × CD3	BFCR4350A	IgG1Fc region	Phase 1	80	Mid 2021	NCT03275103
GPRC5D × CD3	JNJ-64407564	IgG1Fc region	Phase 1	185	Mid 2021	NCT03399799

The majority of BsAbs are effector cell redirecting and most commonly involve αβ T cells via an anti-CD3 arm connected to a tumor antigen binding site. Anti-NKp30 BsAbs, which bind to natural killer (NK) cells, as well as BsAbs engaging CD16A and NKG2D, which bind to NK cells and γδ T cells, also exist in various stages of development (12, 15, 19). Two other types of BsAbs include tumor-targeted immunomodulators and dual immunomodulators. Tumor-targeted immunomodulators direct immune co-stimulation to pre-activated, tumor-infiltrating immune cells (e.g., tumor-specific effector T cells) by binding to a tumor antigen and a costimulatory molecule (e.g., 4-1BB on T cells). By activating a pool of many different tumor-specific T cell clones, rather than harnessing non-specific effector cells to one pre-determined tumor antigen, tumor-targeted immunomodulators may recognize tumor cells with antigen heterogeneity and build immunological memory. Dual immunomodulators bind two separate immunomodulating targets (usually T cell checkpoint pathways such as PD-1, LAG-3, or TIM-3) to block the mechanisms of the immunosuppressive tumor microenvironment (12). Importantly, BsAbs are effective in directing lysis of malignant cells with low antigen expression levels, a significant advantage when targeting surface molecules that are down-regulated as a mode of tumor evasion. Since no immunomodulatory BsAbs are currently in clinical trials for MM, this review will focus on immune cell redirecting BsAbs.

BsAbs can combine multiple functions of individual mAbs (such as direct cancer cell lysis, blocking malignant signaling pathways, independent T cell activation), entailing a comparatively simpler treatment regimen than one requiring the combination of multiple separate agents (12).

**Figure 2:**
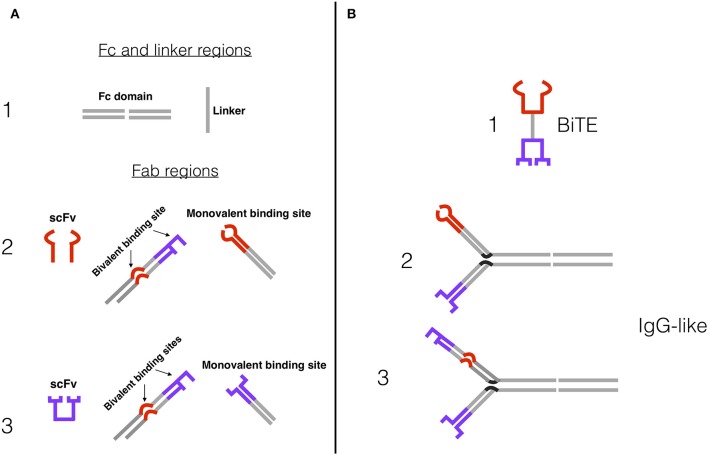
Examples of BsAb constructs and designs. **(A)** (1) Fc domain and peptide linker, which connect antigen-binding sites in IgG-like BsAbs and Fc-less BsAbs, respectively. The Fc domain confers a number of additional features. (2) Effector cell binding sites, usually anti-CD3e for T cells or anti-CD16A for NK cells: single-chain fragment variable (scFv), bivalent binding site (tumor antigen and effector cell binding site), monovalent binding site. (3) Tumor antigen binding sites: scFv, bivalent binding site, monovalent binding site. **(B)** Simplified BsAb constructs. (1) Bispecific T cell Engager (BiTE) design, comprised of two scFvs and a peptide linker—e.g., blinatumomab. (2) Bivalent IgG-like BsAb design, with an Fc domain and two monovalent binding arms. (3) Trivalent IgG-like 2+1 BsAb design, with an Fc domain, a bivalent anti-tumor antigen and anti-CD3 or CD16A arm, and a monovalent anti-tumor antigen arm. Tetravalent BsAbs have a similar construction, but with two bivalent arms.

BsAbs are classed into ~100 different formats, which fall roughly into two categories: BsAbs that include a fragment crystallizable (Fc) region, and BsAbs that consist of only fragment antigen-binding (Fab) variable regions and linkers. Both designs are being tested in MM. Some BsAbs with an Fc region (especially those that target NK cells rather than T cells) exhibit Fc-mediated effector functions, including antibody-dependent cell-mediated cytotoxicity (ADCC) via Fc receptor (FcR) binding, as well as added stability and a longer half-life (48, 53, 54). Anti-CD3 BsAbs often have an engineered, effector-silenced Fc region that mainly imparts a longer half-life and added stability (55). Fc-containing BsAbs are structurally similar to IgG molecules, with variations on the symmetry of their molecular composition and the number of binding sites (Figure 2A) (54). Increasing the number of binding sites on a BsAb (multivalency) can increase target affinity, especially for targets with low expression on tumor cells.

BsAbs that lack an Fc region merely consist of the antigen-binding sites of two antibodies, and most commonly follow the single-chain variable fragment (scFv) design. ScFvs only contain the variable regions of the heavy and light chains and are therefore the simplest iterations of the antigen binding site: They are relatively small and commonly connected by a peptide linker (56, 57). The scFv and linker format is utilized in the construction of bispecific T cell engager molecules (BiTEs), of which blinatumomab and AMG420 are examples (Figure 2B). The simplicity and small size of BiTEs and other scFv BsAbs confer both benefits (e.g., relative ease of adding additional scFvs to create trispecific or trivalent molecules) and drawbacks (e.g., short serum half-lives, decreased efficacy and increased cost by requiring repetitive dosing) (58, 59). Independent T cell activation (inducing cytotoxicity without requiring co-stimulation with CD28 or IL-2) has been observed in BsAbs with and without Fc region (12–19). Proposed mechanisms for this include clustering of the TCR-CD3 complexes to induce signaling in the absence of co-stimulation and the predominance of acting on memory T cells that require less stimulation to become activated (13). The wide array of BsAb structural designs and their advantages and disadvantages have been extensively reviewed by Brinkmann and Kontermann (54).

An alternative to exploiting the cytotoxic potential of T cells for tumor destruction is to redirect and activate NK cells. NK cell redirecting BsAb designs, such as Bispecific NK Engagers (BiKEs) (comprised of two scFvs) and BsAbs with modified Fc regions, are currently in clinical trials for hematologic malignancies; their use for MM is a promising future avenue (60, 61). Since NK cells are the first lymphocyte population to reappear after high-dose chemotherapy, NK cell redirecting BsAbs may be used to eradicate minimal residual disease (MRD) after first-line MM treatment (62). Additionally, NK cell redirecting BsAbs have the potential to be used in combination with other treatments, such as adoptive NK cell transfer after autologous stem cell transplantation (autoSCT).

Their mechanism of action makes BsAbs unique candidates for high-risk MM therapy. High-risk MM patients often display a great degree of intra-tumoral genetic heterogeneity (63); therefore, activating the immune system for broad tumor recognition may be more promising than targeting single genetic lesions. The few studies that have investigated the treatment of patients with high-risk disease failed to conclude that intensification of personalized targeted therapy was significantly beneficial (64, 65). Even therapy regimens containing two autoSCTs (“tandem transplants”) only delay disease progression in high-risk patients rather than cure it (66). The need for new therapies that effectively target high-risk and R/R MM is therefore great, and BsAbs have the potential to fulfill this need.

Several novel treatment approaches like chimeric antigen receptor (CAR) T cell therapies, targeted therapies, and combining mAbs are being implemented for high-risk and R/R MM patients, but BsAb therapies offer numerous advantages. Unlike CAR T cell therapies that have to be individualized by *ex vivo* manipulation of patient-derived T cells, BsAb therapies are “one size fits all” therapies that can be started immediately. BsAbs can be given in incremental doses and interrupted if necessary, so treatment-related toxicities are easier to manage than in CAR T cell therapies. This simplifies treatment regimens and study design/infrastructure and reduces costs (48, 67, 68). Notably, a recent report by Maruta et al. provides a direct comparison between target-reactivity and cross-reactivity of BsAb and CAR T cell models in MM, which showed similar tumor-killing activity, but a delay in CAR T cells relative to BsAbs (69). Additionally, targeted therapies directed at a particular genetic lesion (e.g., bortezomib, palbociclib, encorafenib, etc.) may only eradicate a certain subclone containing that lesion (e.g., the clone present in the diagnostic random iliac crest biopsy), whereas other clones (including disease-driving clones present in focal lesions) are spared (63). In contrast, BsAbs target antigens that are broadly expressed in all malignant plasma cells, such as BCMA, CD38, and CD138, and increase the chances of thoroughly eradicating all malignant clones. mAbs can similarly target tumor antigens, but are unable to directly harness the potent lytic power of T cells to aid in tumor destruction (70). The ADCC functions of mAbs are dependent on Fc functionality, which can be inhibited by alternative Fc glycosylation or Fc receptor polymorphisms, activation of inhibitory receptors, and competition with circulating IgG. BsAbs ensure effector cell involvement via their specific binding arm, guaranteeing the retargeting of effector cells against the malignant cell (47, 71). Thus, BsAbs present an unprecedented opportunity for all MM patients, and particularly those with high-risk and R/R MM for whom standard and targeted therapies have failed.

Despite the novel and promising features of BsAbs, these immunotherapeutics have faced considerable roadblocks on the path to commercial approval and widespread use. For T cell redirecting BsAbs, the activation of large proportions of non-specific T cells can lead to significant toxicity and treatment-related adverse events (AEs) (12). Cytokine release syndrome (CRS) is among the most important AEs of BsAb treatment, with multiple instances recorded in clinical trials of blinatumomab, PF-0683135, CC-93269, and AMG420 (68, 72–74). CRS can present as a variety of symptoms, ranging from influenza-like symptoms to neurotoxicity and multi-organ failure; the recommended treatment depends on its grade of severity (68, 75). Low-grade CRS can be treated symptomatically with antihistamines, antipyretics, and fluids, while high-grade CRS is treated with corticosteroids. Notably, a prophylactic protocol (consisting of dose adjustment and premedication with dexamethasone) for severe CRS was successfully devised to limit severe CRS during blinatumomab trials (68, 76). An additional study with dexamethasone and tocilizumab (anti-IL-6) has reduced CC-93269-induced CRS (77, 78).

NK cell redirecting BsAbs, which operate via FcR mediated cytotoxicity, present an alternative immunotherapy that may result in reduced general toxicity (12, 79, 80). However, to be successful in MM, NK cell redirecting BsAbs must find antigen epitopes that are not subject to competitive interference by serum IgGs (such as the high levels of M-paraprotein displayed in many MM patients) (79). CD16A, a type III FcγR, may be such an antigen (62). Hallmarks of tumor immune evasion, such as heterogenous expression and down-regulation of antigen levels, present obstacles to both T cell and NK cell redirecting BsAbs (79). New constructs, such as multivalent and tri-specific BsAbs, are under investigation as possible responses to these concerns (81–83). These new designs may also be pivotal in reducing toxicity.

## MM Drug Targets for BsAbs

Ideally, BsAb therapeutic targets should be highly expressed on malignant cells and absent or at low levels on other cell types to avoid dose-limiting toxicities (84). Additionally, ideal BsAb targets play an important role in the survival and proliferation of malignant cells, preventing their down-regulation as a mechanism of tumor immune evasion (48). Antigen distribution and content vary both between patients and within a given patient, emphasizing that the success of each drug depends not only on construct but on target expression. So far, there are 24 BsAbs in development against eight MM targets (Table 3). Each MM target and its associated drugs will be discussed below, including ongoing clinical trials and preclinical developments.

### BCMA (B Cell Maturation Antigen)

**Figure 3:**
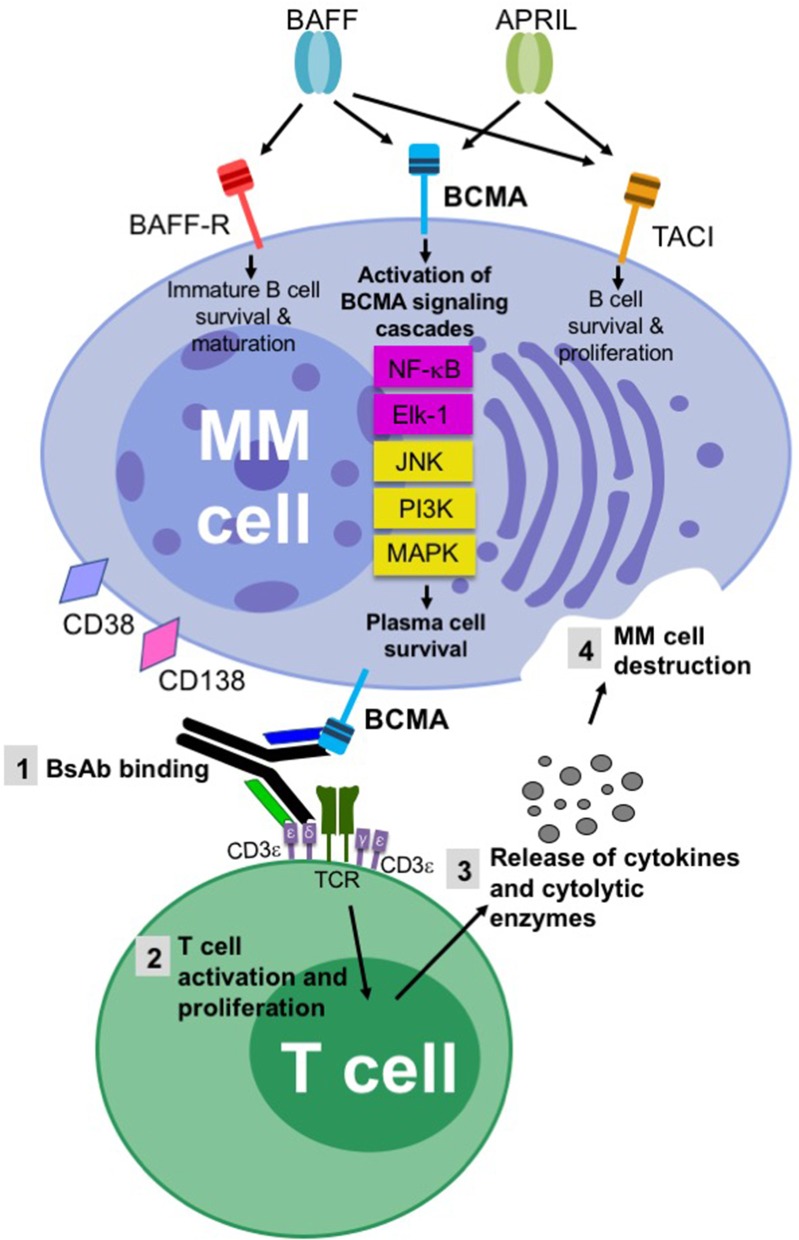
Schematic of key tumor targets and the mechanism of action of BsAbs in multiple myeloma. The superior aspect of the figure highlights the importance of the BCMA/BAFF/APRIL axis and the associated BCMA signaling pathways for malignant plasma cell survival. The inferior aspect of the figure provides a schematic of how BsAbs induce effective T cell-directed MM cell death. A T cell redirecting BsAb binds to BCMA on a MM cell and CD3e on a T cell, coupling these two cells. NK cell redirecting BsAbs bind to CD16A rather than CD3e. Alternative BsAb targets on MM cells include CD38, CD138, FcLR5, CD19, CD319, GPRC5D, and NY-ESO-1. TCR-CD3e cross-linking leads to the activation and proliferation of CD4^+^ and CD8^+^ T cells. Cytokines (i.e., IFN-g, IL-2, IL-6, TNF-a) and cytolytic enzymes (i.e., granzyme B and perforin) are released, resulting in MM cell death.

The most important MM drug target for BsAbs is BCMA (also known as TNFRSF17), which currently has eight BsAbs in clinical development (Table 3) and four in preclinical studies (Table 4). BCMA is a type III transmembrane glycoprotein belonging to the tumor necrosis factor receptor (TNFR) superfamily (90–93). BCMA is expressed primarily on B lineage cells and plays an important role in B cell proliferation (90). It is also widely expressed on all plasma cells, up-regulated during plasma cell differentiation, critical for long-term plasma cell survival, and overexpressed on malignant plasma cells and MM cell lines (90, 94–96) (Figure 3). BCMA is absent on most other cell types, except for low expression on plasmacytoid dendritic cells (pDCs) (90, 97). Importantly, and in contrast to other MM targets such as CD38, BCMA is not expressed on CD34^+^ hematopoietic stem/progenitor cells (90).

**Table 4:** Preclinical models of BsAbs targeting MM.

**Targets**	**Drug**	**Design**	**Model**	**References**
BCMA × CD3	EM801	IgG1 Fc region	MM cell and effector cell co-cultures BMAs of MM patients (autologous T cells) NOG mice with human myeloma allogeneic xenograft Cynomolgus monkeys	Seckinger et al. (85)
BCMA × CD16A	AFM26	Tetravalent, Fc region	NK cell cultures, serum IgG MM cell and primary human NK cell co-cultures MM cell and PBMC co-cultures	Gantke et al. (62)
BCMA × NKp30	CTX-8573	IgG1 Fc region	MM and NK cell co-cultures, in the presence of sBCMA, sBAFF and sAPRIL Humanized mice models engrafted with MM tumors Cynomolgus monkeys	Watkins-Yoon et al. (15)
BCMA × CD3	AP163	Information not available	MM and effector cell co-cultures NSG mice models with human PBMCs and MM or Burkitt lymphoma tumor cells	Li et al. (16)
CD138 × CD3	STL001 or BiTE-hIgFc	scFvs and IgG1 Fc region	MM cell and PBMC co-cultures T cell activation assay NSG mice with human myeloma xenograft	Zou et al. (17)
CD138 × CD3	H-STL002 & M-STL002	scFvs and IgG1 Fc region	MM cell and PBMC co-cultures	Chen et al. (86)
CD38 × CD3	Sorrento CD38/CD3	scFv-Fc region fusion chain and Fab arm	MM cell and PBMC co-cultures NSG mice models with implanted MM or Burkitt lymphoma tumor cells and unstimulated PBMCs	He (87)
CD319 × NKG2D	CS1-NKG2D	BiKE	IL-2 primed NK cultures IL-2 primed PBMC with high, intermediate, low CS1 expression MM cell line co-cultures NSG mice engrafted with human PBMCs and high- and intermediate-CS1 expressing MM cell line xenografts	Chan et al. (19)
GPRC5D × CD3	GPRC5D TRAB	IgG region	MM cell and unstimulated PBMC co-cultures NSG mice model inoculated with human T cells and MM tumor cells NOG mice model engrafted with CD34^+^ hematopoietic stem cells and MM tumor cells	Kodama et al. (88)
NY-ESO-1 × CD3	ImmTAC-NYE	TCR-like HLA-A2/NY-ESO-1_157−165_arm, scFv, peptide linker	MM cell and CD8^+^ cell co-cultures	McCormack et al. (89)
NY-ESO-1 × CD3	A2/NY-ESO-1_157_ –specific BsAb	anti-HLA-A2/NY-ESO-1_157−165_ scFv, scFv, peptide linker	Peripheral blood T cells and T2 cells loaded with NY-ESO-1_157_ peptide co-cultures MM cell and peripheral blood T cell co-cultures Peripheral blood T cells and cells presenting the NY-ESO-1_157−165_ peptide by HLA-A*02:06 co-cultures NOG mice model engrafted with MM cells and activated T cells	Maruta et al. (69)

BCMA has two cognate ligands: (1) B cell activating factor (BAFF, also known as TNFSF13B), which is necessary for B cell development and homeostasis, and (2) a proliferation-inducing ligand (APRIL or TNFSF13A). BAFF and APRIL, either as membranous ligands or in the cleaved, soluble form, bind to BCMA to promote plasma cell growth and survival. Upon ligation by BAFF or APRIL, BCMA activates downstream signaling pathways including the NF-κB, ETS-1 like protein 1 (Elk-1), JNK, ERK, and MAPK pathways (Figure 3) (98–101). This induces pronounced up-regulation of the MCL-1 and BCL-2 anti-apoptotic proteins, preventing dexamethasone-induced cell death. MM patients have up to five times higher soluble BAFF and APRIL serum levels than healthy individuals (102). BCMA also associates with three known TNFR-associated factors (TRAFs)—TRAF1, TRAF2, and TRAF3—which are signal transducers that bind to several members of the TNFR superfamily and facilitate activation of NF-κB, Elk-1, and JNK signaling pathways (101).

Membranous (m)BCMA expression levels per cell increase as healthy plasma cells transform from normal into malignant cells through the disease progression of MGUS to MM (90, 103). Similarly, soluble (s)BCMA levels increase with disease progression, and are found at increased serum levels in MM patients (104). sBCMA levels are also inversely proportional to overall and progression free survival rates (105). sBCMA, which is released from the membrane by spontaneous γ-secretase activity, negatively regulates mBCMA signaling and its associated pathways by competing with mBCMA for BAFF and APRIL (105, 106). Studies suggest that the sequestering of BAFF by sBCMA prevents BAFF from binding to mBCMA and BAFF-R on both healthy and cancer cells. In MM, this blocks the stimulation of normal antibody production, thus contributing to the immunosuppressive MM phenotype by inducing hypogammaglobulinemia (105). Additional studies have shown that high levels of sBCMA may weaken the effectiveness of anti-BCMA type therapies, including BsAbs (104, 107). Additionally, the accumulation of sBCMA in the BM may inhibit BsAb recognition of tumor cells, and reduced mBCMA levels on malignant cells (due to their release as sBCMA by γ-secretase) may further facilitate tumor evasion. These effects may be mitigated by γ-secretase inhibitors (104, 107).

MM patients also display elevated BCMA expression levels on pDCs, which are often in close proximity to MM cells in the BM, and are present in higher numbers in MM patients than in healthy controls (97). pDCs were shown to promote MM progression by secreting factors that enhance MM cell growth (i.e., IL-6), MM chemotaxis (i.e., CXCL-12), and BM angiogenesis (i.e., VEGF), and induce local immunosuppression (i.e., IL-10) (90, 97, 108). Furthermore, pDCs can be resistant to MM therapies, such as bortezomib, lenalidomide, and dexamethasone (97). Given the expression of BCMA on pDCs and their role in MM progression, BCMA-targeting BsAbs have the potential to co-eradicate a key pro-tumorigenic immune cell subset in the BM microenvironment.

Importantly, BCMA seems to be of relatively limited importance to other cell types and tissues. BCMA is not involved in early B cell development or B cell homeostasis, which is in contrast to BAFF receptor (BAFF-R) and Transmembrane Activator and CAML (calcium modulating cyclophilin ligand) Interactor (TACI), which are key to these processes (Figure 3) (102, 109). BCMA-deficient mice develop normally and display healthy physical appearances (110), and only the survival of long-lived BM plasma cells is impaired when compared to wild-type mice (90, 94). In contrast to BCMA, the importance of which to MM is clearly documented, BAFF-R is absent on malignant plasma cells and TACI is expressed at lower levels compared to BCMA (103). Because BCMA's crucial functions in the maintenance and survival of MM cells make its down-regulation unlikely, the likelihood of tumor evasion and drug resistance during treatment is low (85). Collectively, BCMA's plasma cell-specific expression pattern, its overexpression on MM cells and its active involvement in the malignant phenotype make it an ideal BsAb therapeutic target.

#### Clinical Trials of BCMA-Targeting BsAbs

##### PF-06863135 (PF-3135)

PF-06863135 is an anti-BCMA x anti-CD3 BsAb that consists of targeting arms within an IgG2a Fc backbone. It has a half-life of 4–6 days in cynomolgus monkeys (111). PF-06863135 is currently under investigation in a dose-escalation phase 1 trial (ClinicalTrials.gov identifier NCT03269136). The study population includes adult patients with R/R MM, who previously received a proteasome inhibitor, an immunomodulatory drug, and/or an anti-CD38 mAb. Patients received escalating doses of PF-06863135 intravenously once a week to determine the maximum tolerated dose (MTD) and recommended phase 2 dose (RP2D). Results from 23 patients treated weekly over a median duration of 4 weeks showed one complete response, two minimal responses, and nine stable disease cases. Every patient developed more than one AE; most events were grade 1–2, with 5 patients developing grade 3 events. CRS was the most common treatment-related AE, affecting six patients. CRS primarily occurred after the first dose, was dose-dependent and resolved in all patients in less than 4 days. Dose escalation is ongoing as of early 2020, with plans to continue until the maximum tolerated dose is reached (73).

##### TNB-383B

TNB-383B is a trivalent anti-BCMA x anti-CD3 BsAb, with a bivalent anti-BCMA arm (112). The BsAb has a silenced human IgG4 Fc region, with a 10-day half-life in cynomolgus monkeys (113). Preclinical studies testing the drug in BM samples from seven R/R MM patients showed that TNB-383B induced MM cell death, dose-dependent T cell activation, and less cytokine secretion than other BsAbs. NOD/SCID/IL-2Rγ-deficient (NSG) xenograft mouse models showed that TNB-383B reduced tumor growth *in vivo* (114). In June 2019, a phase 1 dose-escalation and expansion trial (NCT03933735) of TNB-383B in patients with R/R MM, who have received at least 3 prior lines of therapy, was initiated. Study arm A will investigate escalating doses of single-agent TNB-383B (25 μg to 40 mg per dose) once every 3 weeks, and arm B will involve an expansion cohort after the recommended dose is established. As of 2019, 12 patients have been enrolled in arm A, with no grade 3 or higher treatment-related AEs (115).

##### REGN5458

REGN5458 is an anti-BCMA x anti-CD3 BsAb, with an Fc domain and anti-BCMA/anti-CD3 Fab domains (116). In preclinical studies, REGN5458 increased surface levels of BCMA on MM cell lines, in addition to inducing T cell killing of MM cells and cytotoxicity in primary human plasma cells (117). In NSG mice, REGN5458 inhibited xenografted tumor growth at doses of 0.4 mg/kg, and at ten times lower doses in immunocompetent mouse models (117). In cynomolgus monkey studies, REGN5458 depleted BCMA^+^ plasma cells in the BM (117). The REGN5458 treatment induced a mild inflammatory response in the cynomolgus monkeys characterized by transiently increased C-reactive protein and serum cytokines, but was otherwise well-tolerated (117). In a comparative study with CAR T cells, REGN5458 displayed targeted cytotoxicity of MM cells lines and primary plasmablasts. Additionally, administration of REGN5458 to NSG mice led to clearance of systemic OPM-2 myeloma tumors within 4 days (compared with CAR T cells, which required 10–14 days for tumor clearance) (117). A clinical trial (NCT03761108) of a first-in-human study of the drug was initiated in January 2019, in patients with R/R MM. The study involves cohorts of multiple REGN5845 dose levels administered intravenously. Results from seven patients after 4 weeks of treatment showed four responses, of which two were MRD negative. Three responders have ongoing responses after a duration of follow-up ranging from 1 to 5.2 months. Every patient had at least one treatment-related AE, five of which were grade 3 or higher (116).

##### REGN5459

REGN5459 is an anti-BCMA x anti-CD3 CD3 BsAb, with an Fc domain and with different binding characteristics from REGN5458 (118). A first-in-human clinical trial (NCT04083534) was initiated in September 2019, in patients with R/R MM. The study involves cohorts of multiple REG5845 dose levels administered intravenously and is expected to end in 2023.

##### CC-93269 (formerly EM901)

CC-93269 is an anti-BCMA × anti-CD3 trivalent BsAb with a bivalent anti-BCMA arm for increased avidity and an IgG1 based Fc region (85, 119, 120). A phase 1 trial (NCT03486067) of a dose-escalation and expansion study of the drug in patients with R/R MM started in April 2018, consisting of intravenous infusion on 28-day cycles (78). As of October 2019, 30 patients had received the drug, with doses ranging from 0.15 to 10 mg. Preliminary results suggest that higher doses (≥3 mg) of CC-93269 result in improved clinical outcomes: overall response rates were 36% in patients treated with 3–6 mg and 89% in patients treated with >6 mg. None of the patients receiving <3 mg responded. The complete response rate was 17% overall, and 44% among the 9 patients treated with 10 mg. The median response rate was 4.1 weeks (range 4.0–13.1), and 92% of responders achieved MRD negativity, often by the end of the first cycle. During follow-up, 29 of the 30 patients experienced at least one treatment-related AE, and 22 patients (73%) experienced a grade 3 or higher AE. The most common treatment-related AEs were neutropenia (43%), anemia (37%), infections (30%), and thrombocytopenia (17%). Twenty-three patients (77%) developed any-grade CRS, including one grade 5 (i.e., death). Most CRS events were successfully managed using dexamethasone and tocilizumab (78).

##### JNJ-64007957

JNJ-64007957 is an anti-BCMA × anti-CD3 BsAb with an IgG1 Fc region (121, 122). In preclinical pharmacokinetic and tolerability studies conducted on cynomolgus monkeys, JNJ-64007957 was well-tolerated at doses up to 10 mg/kg per week. No toxicologically significant effects were found when administered once a week for a 5-week period. The pharmacokinetic report suggested a low anti-drug antibody response, indicating that this drug can be safely administered multiple times per week (123). A phase 1 trial (NCT03145181) of a dose-escalation and expansion study of JNJ-64007957 in patients with R/R MM started in May 2017. The study is being conducted in two parts: one for intravenous administration and one for subcutaneous administration. Additionally, a phase 1 trial (NCT04108195) of subcutaneous daratumumab in combination with intravenous JNJ-64007957 or JNJ-64407564 (i.e., an anti-GPRC5D BsAb) in patients with MM started in January 2020. This study is being conducted in two parts, beginning with a dose escalation phase consisting of 28-day cycles, followed by a dose expansion part. It is expected to end in 2020.

##### AMG420 (formerly BI 836909)

AMG420 is an anti-BCMA × anti-CD3 human BiTE antibody comprised of two scFvs (124). In preclinical studies with co-cultures of unstimulated peripheral blood mononuclear cells (PBMCs) and MM cell lines, AMG420 induced redirected lysis of MM cells and target-dependent release of cytokines by T cells. Anti-tumor activity was further examined in two NSG mouse models reconstituted with human T cells and either subcutaneous or intravenous MM cell line xenotransplantations (125). Comparable dose-dependent anti-tumoral activity was observed in both subcutaneous and intravenous administration regimens of AMG420. Toxicity studies in cynomolgus monkeys showed a dose-dependent decrease of healthy plasma cells in the BM (126). A phase 1 first-in-human dose-escalation and expansion study (NCT02514239) of the drug in patients with R/R MM started in July 2015. Results from this study, which enrolled 42 patients, showed 13 responses, including 6 MRD-negative complete responses, 3 complete responses, and 4 partial responses (127). The median response time was 1 month, and 11 patients responded within the first treatment cycle. Of the 7 patients dosed at 400 μg/d, 5 had complete response with no presence of MRD, and 2 had partial responses. No major toxicities were observed. Thus, 400 μg/d was set as the MTD (128). Of the 42 patients enrolled in this trial, 7 discontinued treatment due to AEs, of which 6 were considered serious, including CRS (3 instances), peripheral polyneuropathy (1 instance), edema (1 instance), and pyrexia (1 instance) (128, 129). Nineteen patients (45%) experienced SAEs, of which infection (14 instances) was most commonly reported (74, 127).

##### AMG701

AMG701 is an anti-BCMA × anti-CD3 human BiTE comprised of two scFvs and an Fc region for extended half-life (~5 days in non-human primates) (14, 130). Preclinical studies of AMG701 in MM cell lines and patient samples showed significant induction of T cell-mediated lysis of MM cells, even at low concentrations and low effector:target cell (E:T) ratios (2:1 and 1:2). This finding was also confirmed in drug-resistant MM cell lines or in the presence of MM-supporting osteoclasts. AMG701 also induced lysis of tumor cells from R/R MM patients in tumor and effector cell co-cultures. Analysis of AMG701-treated MM and effector cell co-cultures revealed that AMG701 induced CD8^+^ and CD4^+^ T cell proliferation (47.5 and 16.7% at 10 ng/ml, respectively) and T cell activation (up-regulation of CD25 and CD69). AMG701 also increased the differentiation of naive CD4^+^ and CD8^+^ T cells toward the central and effector memory phenotype. Additionally, it was postulated that the proliferating T cells potently lysed even those MM cells with reduced BCMA expression. In xenograft MM mouse models, AMG701 blocked tumor growth after 5 days and completely eradicated growth after three injections at all dose levels (0.02, 0.2, and 2 mg/kg); however, with AMG701 treatment alone, mice experienced tumor regrowth by the end of the study. Mice treated with a combination of AMG701 and the immunomodulator and anti-MM agent lenalidomide experienced significant anti-tumor activity 2 days after their first injection, and their tumor volume remained low even after 45+ days of the study. In *in vitro* MM cell and effector cell co-cultures, the AMG701 lenalidomide combination treatment enhanced MM cell-killing as compared to AMG701 treatment alone, including in the presence of MM-supporting cells from the BM microenvironment [such as osteoclasts and bone marrow stromal cells (14, 131)]. A phase 1 trial (NCT03287908) of a dose-escalation and expansion study of the drug in patients with R/R MM started in November 2017 and is expected to end in 2026.

#### Preclinical Models of BCMA-Targeting BsAbs

##### EM801

EM801 is an anti-BCMA × anti-CD3 trivalent BsAb with a bivalent anti-BCMA arm for increased avidity and an IgG1 based Fc region (85). Preclinical studies in human MM cell lines showed that EM801 binds T cells and MM cells, leading to TCR/CD3 cross-linking and activating the CD3 downstream signaling pathways. In BM aspirates from MM patients, EM801 induced significant primary myeloma cell death by autologous T cells, reaching 90% reduction in 48 h (85). To further evaluate the anti-tumor activity of EM801, studies were performed in human MM xenografted immunodeficient mice and in cynomolgus monkeys. In mice, EM801 was potent against highly proliferating MM cells. In monkeys, a reduction in BCMA^+^ plasma cells was observed in the BM and peripheral blood (85).

##### AFM26

AFM26 is an anti-BCMA × anti-CD16A tetravalent BsAb, consisting of a bivalent anti-CD16A arm for increased NK cell avidity connected to a bivalent anti-BCMA arm by an IgG-like backbone (62, 79). AFM26 binds to an epitope of CD16A that is not blocked by serum IgG (e.g., M-protein) binding. A preclinical study conducted on NK cell cultures found that AFM26 exhibits prolonged retention on the surface of NK cells, with receptor retention levels of AFM26 remaining above 60% after 1 h, even in the presence of serum IgG. This is particularly significant for MM because high M-protein levels are characteristic of the disease. In the same preclinical study, AFM26 was applied to primary human NK and MM cell co-cultures (E:T, 5:1), and induced MM cell-specific lysis. In an experiment conducted on multiple MM cell lines with varying BCMA expression levels, AFM26 was found to retain potency even on cells with low BCMA expression. Additionally, unlike mAbs such as daratumumab and elotuzumab (anti-SLAMF7), AFM26 did not induce NK cell lysis. Furthermore, when compared with BiTE incubation on MM and PBMC co-cultures, AFM26 displayed comparable efficacy and markedly reduced cytokine production, suggesting a superior safety profile (62, 132).

##### CTX-8573

CTX-8573 is an anti-BCMA × anti-NKp30 BsAb, with an IgG1-like afucosylated Fc region to additionally engage CD16A on NK cells and γδ T cells (15). It displays a 16-day half-life in cynomolgus monkeys (133). CTX-8573 was tested on human NK and MM cell co-cultures and displayed potent cytotoxicity toward BCMA^+^ tumor cells, including on low BCMA-expressing cell lines. Significantly, this cytotoxic activity was maintained in the presence of sBCMA, sBAFF, and sAPRIL, all of which are displayed at higher levels in high-risk MM patients. A model of the BsAb with an aglycosylated Fc region (to preclude CD16A engagement) retained ADCC, showing that NKp30 engagement alone can promote innate cell activation and cytotoxicity, although CD16A involvement enhances such activity. A preclinical study conducted on multiple humanized mouse models engrafted with MM tumors showed potent anti-tumor activity. Pharmacokinetic and safety profiling in cynomolgus monkeys showed standard biphasic pharmacokinetics and no evidence of systemic immune activation as measured by C-reactive protein levels (133).

##### AP163

AP163 is an anti-BCMA × anti-CD3 BsAb with a 9 h half-life in cynomolgus monkeys (16). Preclinical testing on MM cell lines and effector cell co-cultures showed that AP163 induces cross-linking, T cell activation, cytokine production, proliferation, and redirected target cell killing, eradicating tumor cells. AP163 was studied in multiple NSG mice models injected with human PBMCs and subcutaneous MM cell xenografts or BCMA-expressing Burkitt's lymphoma cell xenografts. In all models, AP163 resulted in T cell activation, cytokine production, and cancer cell killing. In the two MM xenografts tested, AP163 eradicated or significantly delayed tumor growth at doses as low as 0.04 mg/kg. Toxicity testing was carried out on cynomolgus monkeys and non-human primates, in which the drug was well-tolerated at doses up to 5 mg/kg. Significantly, AP163 induced minimal cytokine release as compared to conventional BsAbs (16).

### CD138 (Syndecan-1)

CD138 is a type I transmembrane protein of the syndecan proteoglycan family (134). CD138 has a wide variety of functions, including cell signaling, cell-cell adhesion, cytoskeletal organization, and tumorigenesis (i.e., proliferation, angiogenesis, and metastasis) (135). It is expressed primarily on epithelial cells, transiently on developing mesenchymal cells and at the terminal plasmacytic differentiation stage of B cells (136, 137). Viable MM cells have high expression of membranous (m)CD138; when cells undergo apoptosis, shedding of mCD138 is triggered (138, 139). Studies have found that CD138 suppresses apoptosis in MM cells by activating the insulin-like growth factor-1 receptor; high mCD138 expression can thus indicate non-apoptotic cells, making it an efficient antigen for targeting viable MM cells (140, 141). CD138 also acts as a co-receptor for TACI and APRIL, promoting the APRIL/TACI-associated pathways that induce survival and proliferation of MM cells (142). Additionally, soluble (s)CD138, which is proteolytically shed by matrix metalloproteases and sheddases, is present at high levels in the serum of MM patients and is heavily implicated in disease progression: it acts as a key mediator between MM cells and the BM microenvironment on which they rely, promoting signaling pathways that lead to tumor cell proliferation, angiogenesis, and metastasis. As such, sCD138 is an independent predictor of poor prognosis in MM (143–145).

Various anti-CD138 mAb and T cell engaging MM therapies have taken advantage of the high expression levels of CD138 on MM cells. One mechanism involves coating tumor cells with anti-CD138 mAbs as a method of enhancing dendritic cell cross-presentation of the tumor antigen and the generation of myeloma specific killer T cells (146). CD138's elevated expression on MM cells and its active role in the disease phenotype make it a promising MM BsAb target. Additionally, mCD138's role in preventing apoptosis likely makes tumor cells addicted to this molecule, although a significant proportion of patients were shown to have CD138-negative MM clones (141, 147). Potential drawbacks of CD138 include its high expression on epithelial cells and the accumulation of sCD138 in the BM. In a first-in-human phase 1 trial of an anti-CD138 DM4 (a derivative of the cytotoxic agent maytansine)-antibody conjugate in R/R MM patients, patients suffered from common epithelial-related AEs (e.g., hand-foot syndrome, xerophthalmia, stomatitis, and blurred vision) (148). Furthermore, the characteristic, accelerated shedding of sCD138 and its accumulation in the BM of MM patients may inhibit BsAb recognition of tumor cells. As of February 2020, CD138-targeting MM BsAbs have not yet entered clinical trials.

#### Preclinical Models of CD138-Targeting BsAbs

##### STL001 (also known as BiTE-hIgFc)

STL001 is an anti-CD138 × anti-CD3 BsAb with two scFv arms and an IgG1 Fc region to allow for FcR-mediated NK binding. A preclinical study tested the effects of STL001 on PBMC and MM cell co-cultures (E:T 7:1) and compared the cytotoxicity to that of an anti-CD138 mAb and an anti-CD3 mAb combination and various controls (17). STL001 induced lysis of 90.1% of MM cells after 48 h, compared to 70.5% in the mAb combination and 13.8% and 12.3% in the controls. STL001 was also incubated in a T cell activation assay consisting of PBMCs from healthy donors and IL-2, whereby T cell activation was measured by CD25 and CD69 expression levels. After 24 h, STL001 showed 78.12–85.45% T cell activation efficiency. After 2 weeks of PBMC stimulation and activation, STL001 bound over 96% of the total NK cells. STL001 was also tested at an intravenous dose of 3 mg/kg in an NSG xenograft MM tumor mouse model that had also been injected with unstimulated healthy human PBMCs (E:T, 3:1). Compared to the isotype control, STL001 significantly impaired MM tumor growth, resulting in an ~75% decrease in the mean tumor volume relative to the control (17).

##### H-STL002 and M-STL002

H-STL002 and M-STL002 are anti-CD138 × anti-CD3 BsAbs with two scFv arms and an IgG1 Fc region. A preclinical study tested the effects of these BsAbs on PBMC and MM cell co-cultures (86). After 20 h of incubation, 74–80% of T cells were activated (measured by CD69 expression), and significant MM cell lysis was observed at E:T ratios as low as 7:1. Furthermore, cytotoxicity activity of 98.4% and 98.3% was measured for M-STL002 and H-STL002, respectively (86).

### CD38 (Cyclic ADP Ribose Hydrolase)

CD38 is a type II glycoprotein of the ADP-ribosyl cyclase family, with ectoenzymatic and receptor functionality (149, 150). CD38 plays a regulatory role in calcium homeostasis, nicotinamide adenine dinucleotide (NAD) signaling, and weak adhesion events (151). Originally thought to be a lymphocyte-specific antigen, CD38 was shown to be expressed in nearly every type of tissue, but with elevated expression on hematopoietic cells (151). CD38 is expressed at varying stages of B cell development (i.e., in BM precursor B cells and in terminally differentiated plasma cells) and serves as a marker of T lymphocyte development (149). Additionally, CD38 is uniformly and highly expressed in MM, making it attractive for BsAb targeting. In its role as a receptor, CD38 binds to CD31 (PECAM-1), which is expressed on endothelial cells, lymphoid cells and in the lungs and kidney (152). Interactions between CD38 and CD31 regulate adhesion events between CD38^+^ cells and human umbilical vein endothelial cells. These interactions are also involved in the binding and migration of leukocytes through the endothelial wall, the activation and proliferation of leukocytes, and in B cell development (152, 153). The role of CD38-CD31 interactions is important for MM cell survival in the BM by mediating adhesion to BM endothelial and stromal cells. Clinical studies examining mAb agents that target CD38—such as daratumumab—often lead to down-regulation of CD38 surface expression. Although down-regulation of a target antigen is usually undesirable, in this case it may be beneficial, leading to reduced interaction and support of MM cells by the BM microenvironment (154, 155). A potential obstacle to CD38's use as an MM target is its expression on T cells; however, a preclinical study has shown that T cell fratricide does not preclude the efficacy of anti-CD38 BsAbs as long as tumor cells are lysed at a higher or equal rate to T cells (18).

#### Clinical Trials of CD38-Targeting BsAbs

##### AMG424

AMG424 is an anti-CD38 × anti-CD3 BsAb, with an Fc domain, an anti-CD38 scFv, and an anti-CD3 Fab domain (18). In a preclinical study using MM target cells co-cultured with purified human T cells (E:T 10:1), AMG424 induced complete target cell lysis and limited cytokine release, compared to other BsAbs with higher CD3 affinities. In human PBMC and MM cell line co-cultures (E:T 1:1), AMG424 triggered a pronounced depletion of MM cells and normal B cells, induced a 2-fold increase in T cell numbers and triggered robust T cell activation as measured by induction of CD25. Likewise, in cynomolgus monkeys, intravenous injection of AMG424 triggered T cell activation. However, it also triggered depletion of T cells, B cells, lymphocytes, and monocytes. In an orthotopic tumor model in NSG mice reconstituted with human T cells, intravenous injection of AMG424 induced tumor regression and T cell activation (18). While AMG424 also depleted T cell numbers, the E:T ratio remained stable. A phase 1 first-in-human trial (NCT03445663) of the drug in patients with R/R MM started in July 2018. Part 1 of the study aims to assess the safety and tolerability of AMG424 and determine the MTD and/or biologically active dose. Part 2 will further evaluate the safety and tolerability of the MTD. The trial is expected to end in 2022.

##### GBR1342

GBR1342 is an anti-CD38 × anti-CD3 BsAb, with an Fc domain, an anti-CD38 scFv and an anti-CD3 Fab domain; it has a half-life of ~5 days in rats (156, 157). In human PBMC and MM cell co-cultures (E:T 10:1), GBR1342 demonstrated potent killing of MM cells (157). Additionally, in redirected lysis assays, it demonstrated greater potency than commercial anti-CD38 antibodies, such as daratumumab (157). A phase 1 first-in-human dose-escalation and expansion study (NCT03309111) in patients with previously treated MM began in 2017, with GBR1342 administered by intravenous infusion at an initial dose of 1 ng/kg, with varying dose escalations by cohort (up to 1,000 ng/kg) in 28 day cycles (157). Part 1 of the study is dose evaluating and aimed to assess the safety and tolerability of GBR1342. Part 2 will focus on efficacy exploration. Preliminary results from 19 patients revealed 28 treatment-related AEs in 14 patients, two of which were treatment-related and reversible (i.e., a creatine phosphokinase elevation and an infusion-related reaction, with no neurotoxicity observed to date). Of the 19 patients, 4 were still undergoing treatment with GBR1342 in 2018; as of 2020, the longest duration on the drug has been five cycles, with one patient entering his sixth cycle of dosing at 400 ng/kg (158). In September 2019, GBR1342 was granted orphan drug designation by the FDA (159).

#### Preclinical Models of CD38-Targeting BsAbs

##### Sorrento anti-CD38/CD3 BsAb

Sorrento Therapeutics' anti-CD38/CD3 BsAb has an anti-CD38 Fab arm and an anti-CD3 scFv-Fc region fusion chain. The fusion chain has hinge mutations for reduced Fc region affinity/effector function, to decrease antigen-independent T cell toxicity (87). A preclinical study showed that the BsAb induced potent lysing of CD38^+^ MM cell lines, with antigen density positively correlating with cytotoxic potency. In an *in vivo* follow up, the BsAb construct with the most prolonged anti-tumor activity and best T cell stimulation was the one with a balanced CD38 and CD3 affinity. In a cytotoxicity assay using human PBMCs and MM cell lines, the BsAb showed more potent tumor cell killing than the daratumumab control. In NSG mice models with implanted CD38-expressing Burkitt's lymphoma tumor cells and previously unstimulated human PBMCs, BsAb treatment inhibited tumor growth and prolonged survival. An investigational new drug application is projected to be filed for the BsAb in the first half of 2020 (160).

### CD19

CD19 is a type I transmembrane glycoprotein member of the immunoglobulin superfamily (161). CD19 is primarily involved in the immune response, by modulating B cell receptor (BCR)-dependent and independent signaling to establish B cell signaling thresholds (162–164). It works as the lead receptor in a complex with CD21, CD81, and CD225 to decrease the threshold for receptor-dependent signaling, acting as a co-receptor for BCR signal transduction and interacting with various down-stream protein kinases (including the Src family, Ras family, Abl, Bruton's tyrosine kinase, adapter molecules, and PI3K) (161, 165–169). CD19 is expressed on B cells, from the pre-B cell stage and throughout development, with expression decreasing during terminal plasma cell differentiation (161, 162, 168). CD19 expression is further reduced as plasma cells transform into MM cells. It has been proposed that CD19 loss aids MM cell proliferation (170), and this loss precludes MM patients from benefitting from anti-CD19 therapies. However, there have been reports of R/R MM patients responding to anti-CD19 CAR T cell therapies in combination with other treatments (171, 172). Additionally, super-resolution microscopy has shown very low CD19 expression on MM cells, which was undetectable by flow cytometry but may be accessible to antibodies and modified effector cells (173). These findings make CD19 a potentially interesting BsAb target for MM, despite its unconventional expression pattern and unclear role in the disease phenotype.

#### Clinical Trials of CD19-Targeting BsAbs

##### Blinatumomab

Blinatumomab is an anti-CD19 × anti-CD3 BiTE made of two scFvs, with a half-life of ~2 h in humans (174). In July 2017, it was approved by the FDA for treatment of R/R B cell precursor acute lymphoblastic leukemia (B-ALL) in adults and children (175). Importantly, blinatumomab is the first FDA-approved BsAb. In the phase III trial, which confirmed the clinical benefit of blinatumomab in B-ALL, the drug increased median survival rate from 4 to 7.7 months and resulted in a higher rate of event-free survival than chemotherapy (31% vs. 12%) (TOWER trial, NCT02013167) (176). SAEs including neurologic events, CRS, administration-site reactions, and procedural complications, were reported in 62% of patients treated with blinatumomab as compared to 45% in the chemotherapy group. Results from a phase II study (BLAST, NCT01207388) evaluating blinatumomab in B-ALL found a median OS of 36.5 months after treatment, and more than 50% of patients who achieved MRD after their first cycle were alive at 5 years (72).

Given the success of blinatumomab in B-ALL, it is currently in clinical trials for numerous other B cell malignancies, including R/R MM (177). A phase 1 clinical trial of blinatumomab in combination with salvage autoSCT for patients with R/R MM began in May 2017 (NCT03173430). The study consists of administering up to two 28-day cycles of blinatumomab to patients who previously received high-dose melphalan and autoSCT for MM, with results pending. A case study of blinatumomab-induced response of R/R MM in the context of a secondary pre-B cell ALL emerged in 2017 (172). The patient, a 70-year-old female, developed pre-B-ALL while undergoing lenalidomide therapy for MM, for which she was in partial remission. She underwent cytoreductive therapy and began blinatumomab induction, which resulted in a complete remission of her ALL and a very good partial response of her MM by International Myeloma Working Group criteria (172). Although this case is promising for the application of blinatumomab to other MM patients, it is important to point out that this patient's MM cells stained positive for CD19, which is atypical for MM tumors (172).

### CD319 (SLAMF7 or CS1)

CD319 is a homophilic (self-ligand) surface glycoprotein receptor of the signaling lymphocyte activation molecule (SLAM) family (178). CD319 is a regulatory receptor, with a key role in immune cell function and immune signaling mediation (178, 179). CD319's cytoplasmic tail includes an immunoreceptor tyrosine switch-motif (180). The immunoreceptor tyrosine switch-motif mediates binding to Ewing sarcoma/Friend leukemia integration 1 transcription factor-activated transcript 2 (EAT-2), a member of the SLAM-associated protein (SAP) family of adaptors. CD319/EAT-2 binding determines whether CD319 stimulation will activate or inhibit immune cell functions; in the presence of EAT-2, CD319 plays an activating role, while in the absence of EAT-2, it mediates inhibitory effects (178). CD319 is expressed predominately on NK cells, but also CD8^+^ T cells and B cells, with marked up-regulation during terminal B cell differentiation into plasmablasts and plasma cells (181). It is absent on hematopoietic stem/progenitor cells and blood cancers, except for malignant plasma cells (182, 183). CD319 mRNA has been detected on over 97% of CD138^+^ MM cells, with protein expression confirmed by flow cytometry (184). The function of CD319 in plasma cells and MM cells is not certain: both seemingly lack EAT-2, theoretically suggesting an inhibitory role for CD319 mediation, as is the case in EAT-2-negative NK cells (178, 185). However, a study testing isolated and activated B cells found that stimulation with anti-CD319 mAbs (along with an anti-CD40 mAb and IL-4) increased cell proliferation and induced the expression of growth-supporting cytokines (179). This suggests the possibility of an activating role for CD319 on MM cells, despite their lack of EAT-2. CD319 may also aid in the communication and adhesion between MM cells and the BM microenvironment. A study investigating the effects of an anti-CD319 mAb found that CD319 is localized to the uropod membrane domains of MM cells, regions promoting cell-cell adhesion (183). When CD319 was blocked by the mAb, MM cell adhesion to BM stromal cells was reduced in a dose-dependent manner (183). By supporting adhesion of MM and BM stromal cells, CD319 may promote MM cell proliferation and survival. The ubiquitous and elevated expression of CD319 on MM cells and its possible involvement in disease progression make it a promising BsAb target. As of February 2020, CD319-targeting MM BsAbs have not yet entered clinical trials.

#### Preclinical Models of CD319-Targeting BsAbs

##### CS1-NKG2D BsAb

CS1-NKG2D BsAb is an anti-CD319 × anti-NKG2D bispecific T/NK cell engager made of two scFvs (19). NKG2D is expressed on cytolytic immune cells such as NK cells, CD8^+^ T cells, γδ T cells, and NKT cells (with no expression on CD34^+^ hematopoietic stem/progenitor cells). It is one of the major activating NK cell receptors and a co-stimulatory molecule on cytotoxic CD8^+^ T cells and NKT cells (186). A preclinical study incubated IL-2-primed NK cell cultures with NKG2D and found that the CS1-NKG2D BsAb binds to and triggers the activation of NK cells (19). The study then tested the effects of the BsAb on three different co-cultures consisting of IL-2-primed PBMCs with MM cell lines with high, intermediate, and low CD319 expression. Dose-dependent increases in MM cell lysis were observed in the high and intermediate expression co-cultures. The BsAb was then tested in primary MM patient peripheral blood samples treated with allogeneic PBMCs (E:T, 10:1), which reduced MM cells with high CD319 expression. No specific lysis against T, NKT, or NK cells was found. NSG mice engrafted with human PBMCs and CD319 high- and intermediate-expressing MM cell lines were also administered subcutaneous doses of the BsAb. In this context, only mice engrafted with CD319 high-expressing MM cell lines experienced a significant prolonged survival in response to the BsAb (i.e., ~40 days compared to ~30 days in the control group) (19).

### FcRL5 (Fc Receptor-Like 5)

FcRL5 (CD307) is a membrane protein that is closely related to the Fc receptor family. FcRL5 regulates BCR signaling and binds aggregated IgG (84, 187). It is restricted to B lineage cells, with high expression on mature B cells and plasma cells (187). FcRL5 mRNA is overexpressed in MM cells, and one study found FcRL5 protein expression to be three times higher on MGUS and MM cells than on normal plasma cells (84). Another study found comparable expression levels between normal and malignant plasma cells, but higher expression on plasma cells than on normal B cells (188). Significantly, the FcRL5 gene is located at the chromosomal breakpoint in 1q21, the amplification of which is associated with aggressive MM (189). A study analyzing primary MM biopsies found a significant correlation between FcRL5 mRNA expression and 1q21 gain, suggesting that the 1q21 gain can lead to FcRL5 overexpression in high-risk MM patients (188, 190). Therefore, the development of FcRL5-targeting BsAbs may be especially valuable for high-risk MM patients. One concern about FcRL5 as a BsAb target is its large extra-cellular region: large antigen size (in particular large extracellular regions) and increased distance from the epitope to the target cell membrane can interfere with efficient T cell synapse formation (191). However, constructing an anti-FcRL5 BsAb that targets an epitope on the most membrane-proximal domain of FcRL5's extracellular region is an effective solution to this issue; such a BsAb has displayed promising preclinical results at picomolar concentrations (188).

#### Clinical Trials of FcRL5-Targeting BsAbs

##### BFCR4350A (formerly RO7187797)

BFCR4350A is an anti-FcRL5 × anti-CD3 BsAb with an IgG1 Fc region (188, 192). BFCR4350A's anti-FcRL5 arm is constructed to bind to an epitope chosen for its location on the most membrane-proximal extracellular domain, and its ability to achieve efficient synapse formation. In preclinical studies, BFCR4350A was applied to MM cell and CD8^+^ or CD4^+^ T cell co-cultures, resulting in dose-dependent T cell activation and killing of the MM cells. It also induced robust T cell proliferation, with 95% of the CD8^+^ T cells undergoing up to six cell divisions in 5 days (188). BFCR4350A was then tested on co-cultures of patient-derived BM mononuclear cells (BMMCs) with healthy-donor CD8^+^ T cells and healthy BMMCs. BFCR4350A displayed similarly cytotoxic dose-dependent killing of myeloma BMMCs and of normal plasma cells. The preclinical study also examined the activity of BFCR4350A in humanized NSG mice with transplanted CD34^+^-purified human hematopoietic stem cells. The mice were subcutaneously inoculated with MM cells and later given weekly IV doses of 0.5 mg/kg of BFCR4350A, which resulted in tumor regression in all mice. A study consisting of a single intravenous dose with slow infusion of 1–4 mg/kg of BFCR4350A was conducted in cynomolgus monkeys (188). The treatment resulted in T cell activation, transient T cell decrease, complete depletion of B cells in the spleen and BM, robust dose-dependent depletion of B cells in the lymph nodes, a dose-dependent reduction of IgG levels, and mild cytokine release (188). Collectively, plasma cell and IgG depletion suggest effective BFCR4350A activity in the BM. A second preclinical study, testing the efficacy of single host cell construction of BFCR4350A (i.e., *in vivo* as opposed to *in vitro* assembly), found comparable results between these construction methods (193). A phase 1 dose escalation and expansion trial (NCT03275103) of the drug in patients with R/R MM started in September 2017 (Table 3). The drug is being administered intravenously in 21-day cycles, up to a maximum of 17 cycles or unacceptable toxicity, and the expected primary completion date is 2021.

### GPRC5D (G Protein-Coupled Receptor Class C Group 5 Member D)

GPRC5D is a transmembrane orphan receptor of the G protein-coupled receptor family, whose functions are poorly characterized (194–196). MM patients have high *GPRC5D* mRNA expression in their BM, with low expression in normal tissues (194). GPRC5D is also highly expressed on the surface of MM cells, with lower expression on B and plasma cells and no expression on other hematopoietic cells (88). Due to this expression pattern, GPRC5D is thought to play a key role in MM tumor cell proliferation (197). *GPRC5D* mRNA expression has also been associated with the high-risk cytogenetic events del(13q14) and t(4;14), suggesting its possible role as a prognostic marker (194). Therefore, GPRC5D is an interesting and novel target for MM.

#### Clinical Trials of GPRC5D-Targeting BsAbs

##### JNJ-64407564

JNJ-64407564 is an anti-GPRC5D × anti-CD3 BsAb with an IgG1 Fc region (198, 199). A preclinical study tested JNJ-64407564 in a co-culture of MM cell lines and healthy human T cells (E:T, 5:1), a co-culture of healthy human whole blood and MM cell lines, and a co-culture of BMMCs from MM patients and exogenous healthy human T cells (200). JNJ-64407564 induced MM cell directed cytotoxicity in all co-cultures and dose-dependent T cell proliferation in the MM cell line and healthy human T cell co-culture. T cell activation was observed in both healthy human T cell co-cultures but not in the blood co-culture. JNJ-64407564 was then tested in two NSG mice models with human MM xenografts and human PBMCs; the drug led to significant anti-tumor activity and 100% complete responses in both groups. Testing of the drug in cynomolgus monkeys showed no adverse effects (200). A phase 1 dose-escalation and expansion trial (NCT03399799) in patients with R/R MM began in 2017, with JNJ-64407564 administered by intravenous or subcutaneous injection. Additionally, a phase 1 trial (NCT04108195) testing combinations of daratumumab with JNJ-64407564 (anti-GPRC5D BsAb) or JNJ-64007957 (anti-BCMA BsAb) in MM patients started in January 2020. This study is being conducted in two parts, beginning with a dose escalation phase consisting of 28-day cycles, followed by a dose expansion part, and is expected to end in 2021.

#### Preclinical Models of GPRC5D-Targeting BsAbs

##### GPRC5D TRAB

GPRC5D TRAB (T-cell redirecting antibody) is an anti-GPRC5D × anti-CD3 BsAb with an IgG base. A preclinical study testing four prototypes examined their anti-tumor activity (88). Two prototypes were added to a co-culture of unstimulated human PBMCs and GPRC5D-expressing MM cell lines and to a control of unstimulated human PBMCs and GPRC5D-negative lung cancer cell lines, respectively. Both prototypes induced cytotoxicity against the MM cells but not the lung cancer cells; GPRC5D expression levels on MM cells did not strongly impact cytotoxicity. The effects of two BsAbs were also tested in an NSG mouse model xenografted with human T cells and GPRC5D-expressing MM cell lines and in a NOG mouse model engrafted with human CD34^+^ hematopoietic stem/progenitor cells and xenotransplanted with a MM cell line.

Importantly, these mouse models used MM cell lines that possess t(4;14), a translocation associated with high-risk MM (88, 201). In the NSG model, treatment with 10 mg/kg of the BsAb prototypes led to significant reduction in volume of both tumors as compared to the non-tumor specific control BsAb, curing up to 50% of the mice. In the NOG model, IV treatment with 10 mg/kg of the BsAb prototypes induced tumor regression in 60% of mice. The cytotoxicity of GPRC5D TRAB against these MM models suggest that this molecule may represent a promising treatment candidate for high-risk MM patients.

### NY-ESO-1 (New York Esophageal Squamous Cell Carcinoma 1)

NY-ESO-1 (also known as cancer/testis antigen 1B, CTAG1B) is an immunogenic member of the cancer/testis antigen family—a protein family with germ and cancer cell expression patterns—showing nuclear localization in mesenchymal stem cells and predominately cytoplasmic expression in tumor cells (202, 203). Little is known about the biological function of NY-ESO-1, but its structural features and expression patterns have suggested a role in cell cycle progression and growth, apoptosis, germ cell self-renewal and differentiation, and stem and cancer cell proliferation (203–207). Its expression in healthy tissue is limited to testis and placental cells, but it is expressed in a wide range of tumor types, including MM. NY-ESO-1 expression is particularly high in relapsed patients and patients with cytogenetic abnormalities as defined by gene expression profiling (208). In 335 newly diagnosed MM patients, NY-ESO-1 expression was present in 60% of cases in patients with cytogenetic abnormalities vs. 31% of cases with no abnormalities; this number increased to 100% and 61% at relapse, respectively (208). These findings suggest that NY-ESO-1 expression may correlate with MM clonal evolution.

NY-ESO-1 is highly immunogenic, with the ability to elicit simultaneous humoral and cellular immune responses (208, 209). In MM, antibody responses to NY-ESO-1 have been found to correlate with tumor load and disease progression (208, 210). NY-ESO-1-derived peptides are presented on MHC class I molecules, allowing for T cell recognition (69). Spontaneous CD8^+^ T cell responses (recognizing NY-ESO-1 peptides 157–165 presented on HLA-A2) have been exhibited in MM patients, and laboratory expansion of these T cells has resulted in efficient MM cell killing (208). The HLA-A2/NY-ESO-1_157−165_ peptide complex is therefore being used as a target antigen in the development of NY-ESO-1-targeting BsAbs for MM (69, 89). The tumor-specific expression of NY-ESO-1 and its elevated prevalence in high-risk patients makes it a promising BsAb target; however, its MHC machinery-dependent presentation may result in loss of expression as a means of immune escape. The combination of HLA-A2/NY-ESO-1_157−165_-targeting BsAb therapy with agents that increase the expression of MHC-machinery proteins, such as interferon (IFN)-γ, may be an avenue worth exploring, to reduce the chances of immune escape via HLA-A2 down regulation (211–214). As of February 2020, NYE-ESO-1-targeting MM BsAbs have not yet entered clinical trials.

#### Preclinical Models of NY-ESO-1-Targeting BsAbs

##### ImmTAC-NYE

ImmTAC-NYE (immune-mobilizing monoclonal TCR against cancer) is an anti-NY-ESO-1 × anti-CD3 BsAb, consisting of a TCR-like, anti-HLA-A2/NY-ESO-1_157−165_ arm fused to an anti-CD3 scFv arm via a peptide linker (89, 215). A preclinical study of the BsAb in a co-culture of MM cells and CD8^+^ effector T cells (E:T, 10:1) showed dose-dependent tumor lysis at 0.1–10 nM (89). ImmTAC-NYE was able to bind to cells with a low-density of HLA-A2/peptide complexes, suggesting maintained functionality despite MHC down-regulation. The study also found that ImmTAC-NYE-activated T cells release cytokines IFN-γ, IL-2, and TNF-α, which, in addition to attracting effector immune cells to the tumor site, may spur long-term anti-tumor activity by promoting components of the death receptor pathway in tumor cells, providing an additional mechanism of tumor cell killing even after the BsAb is metabolized (89, 216).

##### A2/NY-ESO-1_*157*_-specific BsAb

A2/NY-ESO-1_157_-specific BsAb is an anti-NY-ESO-1 × anti-CD3 BsAb consisting of an anti-HLA-A2/NY-ESO-1_157−165_ scFv connected to an anti-CD3 scFv via a peptide linker (69). A preclinical study testing the BsAb on a co-culture of peripheral blood T cells and antigen presentation-deficient T2 cells loaded with NY-ESO-1_157_ peptide showed that the BsAb triggered T cell production of IFN-γ, IL-2, and TNF-α. The BsAb only released cytokines in the presence of the NY-ESO-1_157_-loaded cells and did not appear to be activated by CD3 binding alone, indicating reduced general toxicity. The BsAb was also tested in a peripheral blood T cell and MM cell co-culture, where it triggered cytokine production and killing of MM cells. A NOG mouse model engrafted with MM cells and activated T cells showed that 10-μg doses of the BsAb significantly suppressed tumor growth. The cross-reactivity of the BsAb with different HLA-A2 alleles was then tested by incubating it in peripheral blood T cell co-cultures with cells presenting the NY-ESO-1_157−165_ peptide by HLA-A^*^02:06 instead of HLA-A^*^02:01. Levels of reactivity between the two different alleles were comparable, suggesting that the BsAb would be successful in patients with either type of HLA-A2. The study, which also directly compared the BsAb to a CAR T cell construct with the same anti-HLA-A2/NY-ESO-1_157−165_ scFv, found that the anti-tumor effects of the BsAb were seen earlier than those of the CAR T cell therapy. This may be explained by the fact that the cytolytic synapses induced by the BsAb were more similar to those formed by TCR binding to HLA/peptide complexes than the synapses induced by the CAR T cells were (69).

## Future Directions and Conclusions

The development of BsAb treatments for MM has great potential. Preclinical findings in *in vitro* and *in vivo* models have shown effective tumor eradication. Additionally, preliminary clinical results of BCMA-targeting BsAbs PF-06863135 and AMG420 have been promising, with an absence of dose-limiting toxicity in PF-06863135 and six MRD-negative complete responses in AMG420 (73, 74). As more study results materialize, BsAbs will continue to be refined to increase efficacy and safety.

Multiple areas of further development are already emerging, with a focus on reducing treatment-related adverse events and on conquering tumor evasion.

Down-regulation of the target antigen is a classic mechanism of tumor resistance, and multivalent BsAb constructs that increase target avidity, as well as trispecific antibodies that target more than one tumor antigen, may be methods to overcome this obstacle (82). Targeting multiple tumor antigens in one antibody may also prove useful in addressing the heterogeneity of target expression on malignant cells. The increased specificity provided by trispecific antibodies may lead to new combinations of MM targets or to novel targets altogether.

Multi-target specificity may also be a crucial development for avoiding B cell aplasia, leukopenia, and the accompanying increased risk of infection, by ensuring that only the cells that express a particular antigen combination are directly targeted, reducing the chance that healthy cells are lysed. Because many target antigens are also expressed on non-transformed B cells and plasma cells (and are thus targeted by effector T cells), the depletion of B and plasma cell compartments poses a risk of AEs like infection. Febrile neutropenia occurs in up to 40% of B-ALL patients treated with blinatumomab (48, 217). In most patients receiving higher doses of blinatumomab, hypogammaglobulinemia has been observed, but there has been no evidence for an increase in long-term infectious complications. A study examining the long-term effects of CD20-expressing B cell depletion in lymphoma and rheumatoid arthritis patients undergoing rituximab therapy was conducted in 2011. It found that multiple courses of drug exposure may result in IgG and IgM levels below the lower limit of normal serum levels, halted plasma cell formation, and higher serious infection risks (218). B cell aplasia resulting from CAR T cell therapy has been addressed by intravenous or subcutaneous immunoglobulin replacement therapy, and it has been suggested that the same response can be used during BsAb treatment until B cells have recovered (48, 219). Emerging BsAb technologies may prevent against unnecessary B and plasma cell depletion by increasing their specificity. A promising example of such technology is the “split” trispecific antibody, which is divided into two scFv halves, both connected to the same anti-CD3 antibody (83). The CD3-binding site only becomes functional when *both* scFvs have attached to their target antigen, ensuring that effector cell lysis is only directed at cells expressing both antigens. Thus, antigen combinations uniquely expressed by the cancer cells can be targeted, without accompanying B cell and plasma cell depletion (83).

Multi-target specificity is likely to also reduce BsAbs' toxicity profile by limiting the instances of T cell activation. Increasing target avidity may be another way to decrease unwanted cell lysis and the associated risks of aplasia and cytotoxicity. Asymmetric BsAb constructs with bivalent sites for the tumor antigen may not only increase the strength of binding to tumor cells, but also avoid CD3 activation in the absence of sufficient target antigens (e.g., in the case of low antigen expression on healthy cells) (81).

Independent T cell activation, a feature displayed by some but not all BsAbs, is an important area of further development. In some MM cases, inhibition of co-stimulation in the tumor microenvironment via expression of co-inhibitory molecules aids tumor evasion by neutralizing T cell activity (82). BsAb designs that induce biological synergies, resulting in independent T cell activation without the need for costimulatory molecules (e.g., the designs of anti-BCMA BsAb AMG420, anti-CD38 BsAb AMG424, and anti-FcRL5 BsAb BFCR4350A), are thus important for further development (18, 125, 188). Trispecific antibody models designed to stimulate two T cell antigens (rather than only CD3) aim to increase and prolong T cell activation without the need of external co-stimulatory models. One such MM-specific model targets CD38 on the tumor cell, and stimulates both CD3 and CD28 on the T cell (220). CD28, the most important “second signal” on T cells, is also expressed on MM cells at low levels (221, 222). A preclinical study testing the effects of the trispecific antibody was conducted on co-cultures of human PBMCs and MM cell lines and in NSG mouse models xenografted with human CD8^+^ T cells and MM cell lines. The study found that the inclusion of the CD28-binding site not only eliminated the need for external co-stimulation, but also prolonged T cell survival, improved recognition of MM cells, reduced non-specific toxicity, and contributed significantly to anti-tumor efficacy. In the NSG mouse model, tumor growth was completely suppressed in the presence of antibody doses as low as 1 μg/kg (220). These results encourage the further development of trispecific anti-CD28 arm-including antibodies and are particularly promising for MM antibodies, given their increased specificity resulting from MM cell CD28 expression.

Combining BsAbs with immune checkpoint inhibitors (ICIs) may play a key role in the advancement of MM-targeting BsAb therapy by preventing against T cell exhaustion. T cell exhaustion is a feature of MM that may be exacerbated by treatment with BsAbs (223, 224). PD-1/PD-L1 signaling is a hallmark of tumor immunosuppression and T cell exhaustion. Increased PD-1 and PD-L1 expression has been observed in MM patients throughout the course of disease progression, resulting in T cell deactivation and allowing for tumor growth (225). Clinical cases of BsAb-induced T cell exhaustion have been recorded, with a blinatumomab-resistant ALL patient displaying an increase of PD-L1-expressing B-precursor ALL cells (224). Significantly, preclinical findings in MM-targeting BsAbs have also suggested induced T cell exhaustion: increased PD-1 expression in T cells after stimulation by anti-FcRH5/CD3 BsAb in the presence of target-expressing MM cells was observed in cynomolgus monkeys and led to reduced lysis of PD-L1 expressing target-cells (188). However, such mechanisms of T cell exhaustion can be therapeutically countered using ICIs (81). Combinations of anti-PD-1/PD-L1 mAbs with BsAbs have enhanced T cell activation and proliferation and increased cancer cell lysis *in vitro* in multiple studies (226, 227). In MM, the addition of an anti-PD-L1 mAb to anti-FcRH5/CD3 BsAb therapy significantly increased the efficiency of MM cell killing *in vitro* and *in vivo*, restoring T cell activity (188). Such combinations of ICIs with BsAbs may be pivotal in developing treatments that are responsive to immunosuppression.

Combinations of BsAbs with therapeutic Treg depletion may also assist in fighting against immunosuppression. A potential concern about BsAb treatment is that independent T cell activation may also activate unwanted Tregs (82). In MM, Treg numbers are abnormally high, aiding immunosuppression of effector cells in the BM microenvironment (42). Combinations of BsAbs with therapeutic Treg depletion may be helpful or necessary. A preclinical study examining Treg levels in the blood of 42 blinatumomab-treated R/R B-ALL patients confirmed a negative correlation between Treg levels and response to blinatumomab therapy. Importantly, depleting Tregs *in vitro* restored the blinatumomab-triggered proliferation activity of patient T cells. Therapeutic Treg depletion may be achieved *in vivo* by treating patients with cyclophosphamide or fludarabine before blinatumomab therapy (228). Co-infusion of ICIs and manipulation of BsAb design to recruit additional cell types may also prove valuable in overcoming immunosuppression (82). An interesting model of BsAb design ingenuity can be seen in “TriKEs,” which are trispecific killer cell engagers. TriKEs are like NK cell redirecting BsAbs (e.g., BiKEs), with the added integration of IL-15 to drive expansion of NK cells for increased anti-tumoral activity. Preclinical studies in AML have shown TriKE activity to be more efficient than BiKE activity (229). Similarly, innovative BsAb designs such as BsAb-armed T cells are also being applied to MM (230). A clinical study (NCT00938626) targeting myeloma precursor cells in standard and high-risk MM patients administered the patients with anti-CD3 × anti-CD2 BsAb-armed activated T cell infusions prior to autoSCT (230). The infusions induced anti-myeloma IFN-γ and anti-SOX-2 IgG responses, which were then boosted in every patient post-autoSCT. Such responses have been shown to be associated with reduced risk of progression from MGUS to MM (231). This finding suggests that BsAb-armed activated T cell infusions can induce cellular and humoral anti-myeloma immunity that can be detected and boosted after autoSCT (230).

Bispecific immunoconjugates, which consist of two tumor-targeting arms linked to a cytotoxic agent, are another frontier of BsAb innovation with the potential to benefit MM patients. A BsAb-cytokine conjugate−20-C2-2b, which targets tumor antigens CD20 and HLA-DR and is fused to two copies of IFN-α2b—has shown potent inhibition of MM cell lines. A preclinical study found that this compound showed potent cytotoxicity against MM cell lines, even those with limited expression of CD20 or HLA-DR individually (232). As the BsAb field continues to grow and the clinical data accumulates, ongoing innovations will be implemented to improve the immunotherapeutic options for patients with MM and numerous other cancers.

Despite recent therapeutic advances, existing treatments remain largely ineffective for high-risk and R/R MM. Novel immunotherapies, especially BsAbs, provide a new treatment approach for these patients. With numerous phase 1 clinical trials of MM-targeting BsAbs currently underway, the prospect of this new immunotherapeutic treatment for MM patients is on the horizon.

## Author Contributions

CS conceived of the review. CC drafted the manuscript with help from SK. CC and DP created the figures. CC created the tables. DP, CC, and CS edited the manuscript. CC and CS revised the manuscript, and all authors approved its final version.

## Conflict of Interest

The authors declare that the research was conducted in the absence of any commercial or financial relationships that could be construed as a potential conflict of interest.

## References

[1] Robak P Drozdz I Szemraj J Robak T. Drug resistance in multiple myeloma. Cancer Treat Rev. (2018) 70:199–208. 10.1016/j.ctrv.2018.09.001 30245231

[2] Siegel RL Miller KD Jemal A Cancer statistics, 2018. CA Cancer J Clin. (2018) 68:7–30. 10.3322/caac.21442 29313949

[3] Kyle RA Gertz MA Witzig TE Lust JA Lacy MQ Dispenzieri A. Review of 1027 patients with newly diagnosed multiple myeloma. Mayo Clin Proc. (2003) 78:21–33. 10.4065/78.1.21 12528874

[4] Palumbo A Anderson K. Multiple myeloma. N Engl J Med. (2011) 364:1046–60. 10.1056/NEJMra1011442 21410373

[5] Abramson HN. The multiple myeloma drug pipeline-2018: a review of small molecules and their therapeutic targets. Clin Lymphoma Myeloma Leuk. (2018) 18:611–27. 10.1016/j.clml.2018.06.015 30001985

[6] Laubach JP Prada CP Richardson PG Longo DL. Daratumumab, elotuzumab, and the development of therapeutic monoclonal antibodies in multiple myeloma. Clin Pharmacol Ther. (2017) 101:81–8. 10.1002/cpt.550 27806428

[7] Palumbo A Avet-Loiseau H Oliva S Lokhorst HM Goldschmidt H Rosinol L. Revised international staging system for multiple myeloma: a report from international myeloma working group. J Clin Oncol. (2015) 33:2863–9. 10.1200/JCO.2015.61.2267 26240224 PMC4846284

[8] Avet-Loiseau H. Ultra high-risk myeloma. Hematol Am Soc Hematol Educ Program. (2010) 2010:489–93. 10.1182/asheducation-2010.1.489 21239841

[9] Weinhold N Ashby C Rasche L Chavan SS Stein C Stephens OW. Clonal selection and double-hit events involving tumor suppressor genes underlie relapse in myeloma. Blood. (2016) 128:1735–44. 10.1182/blood-2016-06-723007 27516441 PMC5043128

[10] Weinhold N Heuck C Rosenthal A Thanendrarajan S Stein C Van Rhee F. The clinical value of molecular subtyping multiple myeloma using gene expression profiling. Leukemia. (2016) 30:423–30. 10.1038/leu.2015.309 26526987 PMC4740265

[11] Guy DG Uy GL. Bispecific antibodies for the treatment of acute myeloid leukemia. Curr Hematol Malig Rep. (2018) 13:417–25. 10.1007/s11899-018-0472-8 30280288 PMC6295344

[12] Dahlén E Veitonmäki N Norlén P. Bispecific antibodies in cancer immunotherapy. Ther Adv Vaccines Immunother. (2018) 6:3–17. 10.1177/2515135518763280 29998217 PMC5933537

[13] Dreier T Lorenczewski G Brandl C Hoffmann P Syring U Hanakam F. Extremely potent, rapid and costimulation-independent cytotoxic T-cell response against lymphoma cells catalyzed by a single-chain bispecific antibody. Int J Cancer. (2002) 100:690–7. 10.1002/ijc.10557 12209608

[14] Cho S-F Lin L Xing L Liu J Yu T Wen K Anti-BCMA BiTE® AMG 701 potently induces specific T cell lysis of human multiple myeloma (MM) cells and immunomodulation in the bone marrow microenvironment. Blood. (2018) 132:592 10.1182/blood-2018-99-118425

[15] Watkins-Yoon J Guzman W Somanchi A Theriault J Nanjappa P Nelson A CTX-8573, an innate-cell engager targeting BCMA, is a highly potent multispecific antibody for the treatment of multiple myeloma. In: ASH. Available online at: https://ash.confex.com/ash/2019/webprogram/Paper128749.html (accessed January 17, 2020).

[16] Li Z Li Q Zhang G Ma X Li Z Hu X 2020PA Novel bispecific BCMAxCD3 T cell-engaging antibody that treat multiple myeloma (MM) with minimal cytokine secretion. Ann Oncol. (2019) 30(Suppl. 5):797–815. 10.1093/annonc/mdz269.038

[17] Zou J Chen D Zong Y Ye S Tang J Meng H. Immunotherapy based on bispecific T-cell engager with hIgG1 Fc sequence as a new therapeutic strategy in multiple myeloma. Cancer Sci. (2015) 106:512–21. 10.1111/cas.12631 25664501 PMC4452151

[18] Zuch de Zafra CL Fajardo F Zhong W Bernett MJ Muchhal US Moore GL. Targeting multiple myeloma with AMG 424, a novel anti-CD38/CD3 bispecific T-cell–recruiting antibody optimized for cytotoxicity and cytokine release. Clin Cancer Res. (2019) 25:3921–33. 10.1158/1078-0432.CCR-18-2752 30918018

[19] Chan WK Kang S Youssef Y Glankler EN Barrett ER Carter AM. A CS1-NKG2D bispecific antibody collectively activates cytolytic immune cells against multiple myeloma. Cancer Immunol Res. (2018) 6:776–87. 10.1158/2326-6066.CIR-17-0649 29769244 PMC6030494

[20] Appleman LJ Boussiotis VA. T cell anergy and costimulation. Immunol Rev. (2003) 192:161–80. 10.1034/j.1600-065X.2003.00009.x 12670403

[21] FDA. Hematology/Oncology (Cancer) Approvals & Safety Notifications. FDA (2019) Available online at: http://www.fda.gov/drugs/resources-information-approved-drugs/hematologyoncology-cancer-approvals-safety-notifications (accessed November 29, 2019).

[22] List of New Indications and Dosage Forms Drugs.com Available online at: https://www.drugs.com/new-indications.html (accessed November 29, 2019).

[23] Robillard N Avet-Loiseau H Garand R Moreau P Pineau D Rapp M-J. CD20 is associated with a small mature plasma cell morphology and t(11;14) in multiple myeloma. Blood. (2003) 102:1070–1. 10.1182/blood-2002-11-3333 12702507

[24] Kyle RA Durie BGM Rajkumar SV Landgren O Blade J Merlini G. Monoclonal gammopathy of undetermined significance (MGUS) and smoldering (asymptomatic) multiple myeloma: IMWG consensus perspectives risk factors for progression and guidelines for monitoring and management. Leukemia. (2010) 24:1121–7. 10.1038/leu.2010.60 20410922 PMC7020664

[25] Durie-Salmon Staging System International Myeloma Foundation Available online at: https://www.myeloma.org/durie-salmon-staging (accessed July 31, 2019).

[26] Greipp PR Miguel JS Durie BGM Crowley JJ Barlogie B Bladé J. International staging system for multiple myeloma. JCO. (2005) 23:3412–20. 10.1200/JCO.2005.04.242 15809451

[27] Shaughnessy JD Zhan F Burington BE Huang Y Colla S Hanamura I. A validated gene expression model of high-risk multiple myeloma is defined by deregulated expression of genes mapping to chromosome 1. Blood. (2007) 109:2276–84. 10.1182/blood-2006-07-038430 17105813

[28] Boyd KD Ross FM Chiecchio L Dagrada G Konn ZJ Tapper WJ. A novel prognostic model in myeloma based on co-segregating adverse FISH lesions and the ISS: analysis of patients treated in the MRC Myeloma IX trial. Leukemia. (2012) 26:349–55. 10.1038/leu.2011.204 21836613 PMC4545515

[29] Mikhael JR Dingli D Roy V Reeder CB Buadi FK Hayman SR. Management of newly diagnosed symptomatic multiple myeloma: updated mayo stratification of myeloma and risk-adapted therapy (mSMART) consensus guidelines 2013. Mayo Clinic Proceed. (2013) 88:360–76. 10.1016/j.mayocp.2013.01.019 23541011

[30] Chng WJ Dispenzieri A Chim C-S Fonseca R Goldschmidt H Lentzsch S. IMWG consensus on risk stratification in multiple myeloma. Leukemia. (2014) 28:269–77. 10.1038/leu.2013.247 23974982

[31] Treatment Guidelines mSMART. Available online at: https://www.msmart.org/mm-treatment-guidelines (accessed August 21, 2019).

[32] Rajkumar SV. Multiple myeloma: 2016 update on diagnosis, risk-stratification and management. Am J Hematol. (2016) 91:719–34. 10.1002/ajh.24402 27291302 PMC5291298

[33] Sonneveld P Avet-Loiseau H Lonial S Usmani S Siegel D Anderson KC. Treatment of multiple myeloma with high-risk cytogenetics: a consensus of the international myeloma working group. Blood. (2016) 127:2955–62. 10.1182/blood-2016-01-631200 27002115 PMC4920674

[34] Pawlyn C Morgan GJ. Evolutionary biology of high-risk multiple myeloma. Nat Rev Cancer. (2017) 17:543–56. 10.1038/nrc.2017.63 28835722

[35] Rasche L Angtuaco EJ Alpe TL Gershner GH McDonald JE Samant RS. The presence of large focal lesions is a strong independent prognostic factor in multiple myeloma. Blood. (2018) 132:59–66. 10.1182/blood-2018-04-842880 29784643 PMC6034645

[36] Manier S Salem KZ Park J Landau DA Getz G Ghobrial IM. Genomic complexity of multiple myeloma and its clinical implications. Nat Rev Clin Oncol. (2017) 14:100–13. 10.1038/nrclinonc.2016.122 27531699

[37] Kuehl WM Bergsagel PL. Molecular pathogenesis of multiple myeloma and its premalignant precursor. J Clin Invest. (2012) 122:3456–63. 10.1172/JCI61188 23023717 PMC3461901

[38] Cho S-F Anderson KC Tai Y-T. Targeting B Cell Maturation Antigen (BCMA) in multiple myeloma: potential uses of BCMA-based immunotherapy. Front Immunol. (2018) 9:1824. 10.3389/fimmu.2018.01821 30147690 PMC6095983

[39] van Nieuwenhuijzen N Spaan I Raymakers R Peperzak V. From MGUS to multiple myeloma, a paradigm for clonal evolution of premalignant cells. Cancer Res. (2018) 78:2449–56. 10.1158/0008-5472.CAN-17-3115 29703720

[40] Urashima M Ogata A Chauhan D Vidriales MB Teoh G Hoshi Y. Interleukin-6 promotes multiple myeloma cell growth via phosphorylation of retinoblastoma protein. Blood. (1996) 88:2219–27. 10.1182/blood.V88.6.2219.bloodjournal8862219 8822942

[41] Hayashi T Hideshima T Nguyen AN Munoz O Podar K Hamasaki M. Transforming growth factor beta receptor I kinase inhibitor down-regulates cytokine secretion and multiple myeloma cell growth in the bone marrow microenvironment. Clin Cancer Res. (2004) 10:7540–6. 10.1158/1078-0432.CCR-04-0632 15569984

[42] Bryant C Suen H Brown R Yang S Favaloro J Aklilu E. Long-term survival in multiple myeloma is associated with a distinct immunological profile, which includes proliferative cytotoxic T-cell clones and a favourable Treg/Th17 balance. Blood Cancer J. (2013) 3:e148. 10.1038/bcj.2013.34 24036947 PMC3789202

[43] Muthu Raja KR Rihova L Zahradova L Klincova M Penka M Hajek R. Increased T regulatory cells are associated with adverse clinical features and predict progression in multiple myeloma. PLoS ONE. (2012) 7:e47077. 10.1371/journal.pone.0047077 23071717 PMC3468567

[44] Hoyos V Borrello I. The immunotherapy era of myeloma: monoclonal antibodies, vaccines, and adoptive T-cell therapies. Blood. (2016) 128:1679–87. 10.1182/blood-2016-05-636357 27506540

[45] Staerz UD Kanagawa O Bevan MJ. Hybrid antibodies can target sites for attack by T cells. Nature. (1985) 314:628–31. 10.1038/314628a0 2859527

[46] Perez P Hoffman RW Shaw S Bluestone JA Segal DM. Specific targeting of cytotoxic T cells by anti-T3 linked to anti-target cell antibody. Nature. (1985) 316:354–6. 10.1038/316354a0 3160953

[47] Chames P Baty D. Bispecific antibodies for cancer therapy: the light at the end of the tunnel? MAbs. (2009) 1:539–47. 10.4161/mabs.1.6.10015 20073127 PMC2791310

[48] Velasquez MP Bonifant CL Gottschalk S. Redirecting T cells to hematological malignancies with bispecific antibodies. Blood. (2018) 131:30–8. 10.1182/blood-2017-06-741058 29118005 PMC5755042

[49] Linke R Klein A Seimetz D. Catumaxomab. MAbs. (2010) 2:129–36. 10.4161/mabs.2.2.11221 20190561 PMC2840231

[50] Approved antibodies The Antibody Society Available online at: https://www.antibodysociety.org/resources/approved-antibodies/ (accessed August 15, 2019).

[51] Franchini M Marano G Pati I Candura F Profili S Veropalumbo E. Emicizumab for the treatment of haemophilia A: a narrative review. Blood Transfus. (2019) 17:223–8. 10.2450/2019.0026-19 31246563 PMC6596376

[52] Labrijn AF Janmaat ML Reichert JM Parren PWHI. Bispecific antibodies: a mechanistic review of the pipeline. Nat Rev Drug Discov. (2019) 18:585–608. 10.1038/s41573-019-0028-1 31175342

[53] Kontermann RE Brinkmann U. Bispecific antibodies. Drug Discov Today. (2015) 20:838–47. 10.1016/j.drudis.2015.02.008 25728220

[54] Brinkmann U Kontermann RE. The making of bispecific antibodies. mAbs. (2017) 9:182–212. 10.1080/19420862.2016.1268307 28071970 PMC5297537

[55] Liu H Saxena A Sidhu SS Wu D. Fc engineering for developing therapeutic bispecific antibodies and novel scaffolds. Front Immunol. (2017) 8:38. 10.3389/fimmu.2017.00038 28184223 PMC5266686

[56] Huston JS Levinson D Mudgett-Hunter M Tai MS Novotný J Margolies MN. Protein engineering of antibody binding sites: recovery of specific activity in an anti-digoxin single-chain Fv analogue produced in *Escherichia coli*. Proc Natl Acad Sci USA. (1988) 85:5879–83. 10.1073/pnas.85.16.5879 3045807 PMC281868

[57] Ahmad ZA Yeap SK Ali AM Ho WY Alitheen NBM Hamid M. scFv antibody: principles and clinical application. Clin Dev Immunol. (2012) 2012:980250. 10.1155/2012/980250 22474489 PMC3312285

[58] Schubert I Kellner C Stein C Kügler M Schwenkert M Saul D. A single-chain triplebody with specificity for CD19 and CD33 mediates effective lysis of mixed lineage leukemia cells by dual targeting. MAbs. (2011) 3:21–30. 10.4161/mabs.3.1.14057 21081841 PMC3038008

[59] Klinger M Brandl C Zugmaier G Hijazi Y Bargou RC Topp MS. Immunopharmacologic response of patients with B-lineage acute lymphoblastic leukemia to continuous infusion of T cell–engaging CD19/CD3-bispecific BiTE antibody blinatumomab. Blood. (2012) 119:6226–33. 10.1182/blood-2012-01-400515 22592608

[60] Wu J Fu J Zhang M Liu D. AFM13: a first-in-class tetravalent bispecific anti-CD30/CD16A antibody for NK cell-mediated immunotherapy. J Hematol Oncol. (2015) 8:96. 10.1186/s13045-015-0188-3 26231785 PMC4522136

[61] Barrow AD Colonna M. Exploiting NK cell surveillance pathways for cancer therapy. Cancers. (2019) 11:55. 10.3390/cancers11010055 30626155 PMC6356551

[62] Gantke T Reusch U Kellner C Ellwanger K Fucek I Weichel M AFM26 is a novel, highly potent BCMA/CD16A-directed bispecific antibody for high affinity NK-cell engagement in multiple myeloma. JCO. (2017) 35:8045 10.1200/JCO.2017.35.15_suppl.8045

[63] Schürch CM Rasche L Frauenfeld L Weinhold N Fend F. A review on tumor heterogeneity and evolution in multiple myeloma: pathological, radiological, molecular genetics, and clinical integration. Virchows Arch. (2019) 476:337–51. 10.1007/s00428-019-02725-3 31848687

[64] Jethava Y Mitchell A Zangari M Waheed S Schinke C Thanendrarajan S Dose-dense and less dose-intense total therapy 5 for gene expression profiling-defined high-risk multiple myeloma. Blood Cancer J. (2016) 6:e453 10.1038/bcj.2016.64 27471869 PMC5030385

[65] Jethava YS Mitchell A Epstein J Zangari M Yaccoby S Tian E. Adverse metaphase cytogenetics can be overcome by adding bortezomib and thalidomide to fractionated melphalan transplants. Clin Cancer Res. (2017) 23:2665–72. 10.1158/1078-0432.CCR-15-2620 27810902 PMC6080620

[66] Cavo M Goldschmidt H Rosinol L Pantani L Zweegman S Salwender HJ Double vs single autologous stem cell transplantation for newly diagnosed multiple myeloma: long-term follow-up (10-years) analysis of randomized phase 3 studies. Blood. (2018) 132:124 10.1182/blood-2018-99-112899

[67] Cohen AD Raje N Fowler JA Mezzi K Scott EC Dhodapkar MV. How to train your T cells: overcoming immune dysfunction in multiple myeloma. Clin Cancer Res. (2019) 26:1541–54. 10.1158/1078-0432.CCR-19-2111 31672768 PMC8176627

[68] Shimabukuro-Vornhagen A Gödel P Subklewe M Stemmler HJ Schlößer HA Schlaak M. Cytokine release syndrome. J Immunother Cancer. (2018) 6:56. 10.1186/s40425-018-0343-9 29907163 PMC6003181

[69] Maruta M Ochi T Tanimoto K Asai H Saitou T Fujiwara H. Direct comparison of target-reactivity and cross-reactivity induced by CAR- and BiTE-redirected T cells for the development of antibody-based T-cell therapy. Sci Rep. (2019) 9:13293. 10.1038/s41598-019-49834-2 31527633 PMC6746725

[70] Sedykh SE Prinz VV Buneva VN Nevinsky GA. Bispecific antibodies: design, therapy, perspectives. Drug Des Dev Ther. (2018) 12:195–208. 10.2147/DDDT.S151282 29403265 PMC5784585

[71] Chames P Van Regenmortel M Weiss E Baty D. Therapeutic antibodies: successes, limitations and hopes for the future. Br J Pharmacol. (2009) 157:220–33. 10.1111/j.1476-5381.2009.00190.x 19459844 PMC2697811

[72] Amgen Announces BLINCYTO® (blinatumomab) Five-Year Overall Survival Data at EHA 2019 (2009). Available online at: https://finance.yahoo.com/news/amgen-announces-blincyto-blinatumomab-five-060000341.html (accessed August 24, 2019).

[73] Noopur S Raje Andrzej Jakubowiak Cristina Gasparetto Robert F Cornell Heike I Krupka Daniel Navarro Safety, clinical activity, pharmacokinetics, and pharmacodynamics from a phase i study of PF-06863135, a B-Cell Maturation Antigen (BCMA)-CD3 bispecific antibody, in patients with relapsed/refractory multiple myeloma. Blood. 134(Suppl. 1):1869 10.1182/blood-2019-121805

[74] ajones AMG 420 Continues to Show Efficacy in Relapsed/Refractory Myeloma. ASH Clinical News. (2019) Available online at: https://www.ashclinicalnews.org/multiple-myeloma/amg-420-continues-show-efficacy-relapsed-refractory-myeloma/ (Accessed January 26, 2020).

[75] Khadka RH Sakemura R Kenderian SS Johnson AJ. Management of cytokine release syndrome: an update on emerging antigen-specific T cell engaging immunotherapies. Immunotherapy. (2019) 11:851–7. 10.2217/imt-2019-0074 31161844

[76] Topp MS Gökbuget N Stein AS Zugmaier G O'Brien S Bargou RC Safety and activity of blinatumomab for adult patients with relapsed or refractory B-precursor acute lymphoblastic leukaemia: a multicentre, single-arm, phase 2 study. Lancet Oncol. (2015) 16:57–66. 10.1016/S1470-2045(14)71170-2 25524800

[77] Einsele H Rasche L Topp MS Martin Kortüm K Duell J. The use of bispecific antibodies to optimize the outcome of patients with acute leukemia, lymphoma and multiple myeloma after SCT. Bone Marrow Transplant. (2019) 54:721–6. 10.1038/s41409-019-0596-z 31431702

[78] Ahle S Early-phase trial suggests bispecific antibody CC-93269 has activity in relapsed/refractory multiple myeloma. ASH Clin News. (2020) Available online at: https://www.ashclinicalnews.org/on-location/ash-annual-meeting/early-phase-trial-suggests-cc-93269-activity-relapsed-refractory-multiple-myeloma/ (accessed January 25, 2020).

[79] Ellwanger K Reusch U Fucek I Wingert S Ross T Müller T. Redirected optimized cell killing (ROCK®): a highly versatile multispecific fit-for-purpose antibody platform for engaging innate immunity. MAbs. (2019) 11:899–918. 10.1080/19420862.2019.1616506 31172847 PMC6601565

[80] Rothe A Sasse S Topp MS Eichenauer DA Hummel H Reiners KS A phase 1 study of the bispecific anti-CD30/CD16A antibody construct AFM13 in patients with relapsed or refractory hodgkin lymphoma. Blood. (2015) 125:4024 10.1182/blood-2014-12-614636 25887777 PMC4528081

[81] Kobold S Pantelyushin S Rataj F vom Berg J. Rationale for combining bispecific T cell activating antibodies with checkpoint blockade for cancer therapy. Front Oncol. (2018) 8:285. 10.3389/fonc.2018.00285 30090763 PMC6068270

[82] Duell J Lammers PE Djuretic I Chunyk AG Alekar S Jacobs I. Bispecific antibodies in the treatment of hematologic malignancies. Clin Pharmacol Ther. (2019) 106:781–91. 10.1002/cpt.1396 30770546 PMC6766786

[83] Banaszek A Bumm TGP Nowotny B Geis M Jacob K Wölfl M. On-target restoration of a split T cell-engaging antibody for precision immunotherapy. Nat Commun. (2019) 10:5387. 10.1038/s41467-019-13196-0 31772172 PMC6879491

[84] Elkins K Zheng B Go M Slaga D Du C Scales SJ. FcRL5 as a target of antibody–drug conjugates for the treatment of multiple myeloma. Mol Cancer Ther. (2012) 11:2222–32. 10.1158/1535-7163.MCT-12-0087 22807577

[85] Seckinger A Delgado JA Moser S Moreno L Neuber B Grab A. Target expression, generation, preclinical activity, and pharmacokinetics of the BCMA-T cell bispecific antibody EM801 for multiple myeloma treatment. Cancer Cell. (2017) 31:396–410. 10.1016/j.ccell.2017.02.002 28262554

[86] Chen D Zou J Zong Y Meng H An G Yang L. Anti-human CD138 monoclonal antibodies and their bispecific formats: generation and characterization. Immunopharmacol Immunotoxicol. (2016) 38:175–83. 10.3109/08923973.2016.1153110 26954291

[87] He X Preclinical characterization of an ANTI-CD38/CD3 T CELL-redirecting bispecific antibody. In: ASH. Available online at: https://ash.confex.com/ash/2019/webprogram/Paper131540.html (accessed January 26, 2020).

[88] Kodama T Kochi Y Nakai W Mizuno H Baba T Habu K. Anti-GPRC5D/CD3 bispecific T-cell-redirecting antibody for the treatment of multiple myeloma. Mol Cancer Ther. (2019) 18:1555–64. 10.1158/1535-7163.MCT-18-1216 31270154

[89] McCormack E Adams KJ Hassan NJ Kotian A Lissin NM Sami M. Bi-specific TCR-anti CD3 redirected T-cell targeting of NY-ESO-1- and LAGE-1-positive tumors. Cancer Immunol Immunother. (2013) 62:773–85. 10.1007/s00262-012-1384-4 23263452 PMC3624013

[90] Tai Y-T Anderson KC. Targeting B-cell maturation antigen in multiple myeloma. Immunotherapy. (2015) 7:1187–99. 10.2217/imt.15.77 26370838 PMC4976846

[91] Madry C. The characterization of murine BCMA gene defines it as a new member of the tumor necrosis factor receptor superfamily. Int Immunol. (1998) 10:1693–702. 10.1093/intimm/10.11.1693 9846698

[92] Locksley RM Killeen N Lenardo MJ. The TNF and TNF receptor superfamilies: integrating mammalian biology. Cell. (2001) 104:487–501. 10.1016/S0092-8674(01)00237-9 11239407

[93] Gras M-P Laâbi Y Linares-Cruz G Blondel M-O Rigaut J-P Brouet J-C. BCMAp: an integral membrane protein in the golgi apparatus of human mature B lymphocytes. Int Immunol. (1995) 7:1093–106. 10.1093/intimm/7.7.1093 8527407

[94] O'Connor BP Raman VS Erickson LD Cook WJ Weaver LK Ahonen C. BCMA is essential for the survival of long-lived bone marrow plasma cells. J Exp Med. (2004) 199:91–8. 10.1084/jem.20031330 14707116 PMC1887725

[95] Avery DT Kalled SL Ellyard JI Ambrose C Bixler SA Thien M. BAFF selectively enhances the survival of plasmablasts generated from human memory B cells. J Clin Invest. (2003) 112:286–97. 10.1172/JCI18025 12865416 PMC164292

[96] Chiu A Xu W He B Dillon SR Gross JA Sievers E. Hodgkin lymphoma cells express TACI and BCMA receptors and generate survival and proliferation signals in response to BAFF and APRIL. Blood. (2007) 109:729–39. 10.1182/blood-2006-04-015958 16960154 PMC1785096

[97] Chauhan D Singh AV Brahmandam M Carrasco R Bandi M Hideshima T. Functional interaction of plasmacytoid dendritic cells with multiple myeloma cells: a therapeutic target. Cancer Cell. (2009) 16:309–23. 10.1016/j.ccr.2009.08.019 19800576 PMC2762396

[98] Bossen C Tardivel A Willen L Fletcher CA Perroud M Beermann F. Mutation of the BAFF furin cleavage site impairs B-cell homeostasis and antibody responses. Eur J Immunol. (2011) 41:787–97. 10.1002/eji.201040591 21287546

[99] Rennert P Schneider P Cachero TG Thompson J Trabach L Hertig S. A soluble form of B cell maturation antigen, a receptor for the tumor necrosis factor family member april, inhibits tumor cell growth. J Exp Med. (2000) 192:1677–84. 10.1084/jem.192.11.1677 11104810 PMC2193103

[100] Schneider P MacKay F Steiner V Hofmann K Bodmer J-L Holler N. BAFF, a novel ligand of the tumor necrosis factor family, stimulates B cell growth. J Exp Med. (1999) 189:1747–56. 10.1084/jem.189.11.1747 10359578 PMC2193079

[101] Hatzoglou A Roussel J Bourgeade M-F Rogier E Madry C Inoue J TNF receptor family member BCMA (B cell maturation) associates with TNF receptor-associated factor (TRAF) 1, TRAF2, and TRAF3 and activates NF-κB, Elk-1, c-Jun N-terminal kinase, and p38 mitogen-activated protein kinase. J Immunol. (2000) 165:1322–30. 10.4049/jimmunol.165.3.1322 10903733

[102] Moreaux J Legouffe E Jourdan E Quittet P Rème T Lugagne C. BAFF and APRIL protect myeloma cells from apoptosis induced by interleukin 6 deprivation and dexamethasone. Blood. (2004) 103:3148–57. 10.1182/blood-2003-06-1984 15070697 PMC2387243

[103] Sanchez E Li M Kitto A Li J Wang CS Kirk DT. Serum B-cell maturation antigen is elevated in multiple myeloma and correlates with disease status and survival. Br J Haematol. (2012) 158:727–38. 10.1111/j.1365-2141.2012.09241.x 22804669

[104] Chen H Li M Sanchez E Wang CS Udd KA Soof CM Gene expression of Gamma Secretase (GS) complex-related proteins, the enzyme that sheds B-Cell maturation antigen (BCMA), among patients with multiple myeloma (MM) and effects of the GS inhibitor LSN424354 on solubilized bcma in MM and chronic lymphocytic leukemia. Blood. (2016) 128:5641 10.1182/blood.V128.22.5641.5641

[105] Sanchez E Gillespie A Tang G Ferros M Harutyunyan NM Vardanyan S. Soluble B-cell maturation antigen mediates tumor-induced immune deficiency in multiple myeloma. Clin Cancer Res. (2016) 22:3383–97. 10.1158/1078-0432.CCR-15-2224 26960399

[106] Laurent SA Hoffmann FS Kuhn P-H Cheng Q Chu Y Schmidt-Supprian M. γ-secretase directly sheds the survival receptor BCMA from plasma cells. Nat Commun. (2015) 6:7333. 10.1038/ncomms8333 26065893 PMC4490565

[107] Pont MJ Hill T Cole GO Abbott JJ Kelliher J Salter AI. γ-secretase inhibition increases efficacy of BCMA-specific chimeric antigen receptor T cells in multiple myeloma. Blood. (2019) 134:1585–97. 10.1182/blood.2019000050 31558469 PMC6871311

[108] Hideshima T Mitsiades C Tonon G Richardson PG Anderson KC. Understanding multiple myeloma pathogenesis in the bone marrow to identify new therapeutic targets. Nat Rev Cancer. (2007) 7:585–98. 10.1038/nrc2189 17646864

[109] Mackay F Figgett WA Saulep D Lepage M Hibbs ML. B-cell stage and context-dependent requirements for survival signals from BAFF and the B-cell receptor. Immunol Rev. (2010) 237:205–25. 10.1111/j.1600-065X.2010.00944.x 20727038

[110] Xu S Lam K-P. B-cell maturation protein, which binds the tumor necrosis factor family members BAFF and APRIL, is dispensable for humoral immune responses. Mol Cell Biol. (2001) 21:4067–74. 10.1128/MCB.21.12.4067-4074.2001 11359913 PMC87068

[111] Lesokhin AM Raje N Gasparetto CJ Walker J Krupka HI Joh T A phase I, open-label study to evaluate the safety, pharmacokinetic, pharmacodynamic, and clinical activity of PF-06863135, a B-Cell maturation antigen/CD3 bispecific antibody, in patients with relapsed/refractory advanced multiple myeloma. Blood. (2018) 132:3229 10.1182/blood-2018-99-110427

[112] Buelow B Pham D Clarke S Ogana H Dang K Pratap P Development of a fully human T cell engaging bispecific antibody for the treatment of multiple myeloma. J Clin Oncol. 35(15 Suppl.):8017 10.1200/JCO.2017.35.15_suppl.8017

[113] Boyle EM Davies FE Leleu X Morgan GJ. Understanding the multiple biological aspects leading to myeloma. Haematologica. (2014) 99:605–12. 10.3324/haematol.2013.097907 24688108 PMC3971069

[114] Melao Potential Myeloma Treatment, TNB-383B, Makes Progress in New Deal. Myeloma Research News. (2019) Available online at: https://myelomaresearchnews.com/2019/02/20/abbvie-teneobio-to-develop-market-tnb-383b-antibody-for-multiple-myeloma/ (accessed July 5, 2019).

[115] Buelow B D'Souza A Rodriguez C Vij R Nath R Snyder M TNB383B.001: a multicenter, phase 1, open-label, dose-escalation and expansion study of TNB-383B, a bispecific antibody targeting BCMA in subjects with relapsed or refractory multiple myeloma. In: American Society of Hematology 61st Annual Meeting and Exposition. Poster abstract #1874 (2019).

[116] Cooper D Madduri D Lentzsch S Jagannath S Li J Boyapati A Safety and preliminary clinical activity of REGN5458, an anti-Bcma x Anti-CD3 bispecific antibody, in patients with relapsed/refractory multiple myeloma. Blood. (2019) 134:3176 10.1182/blood-2019-126818

[117] Dilillo DJ Olson K Mohrs K Meagher TC Bray K Sineshchekova O REGN5458, a bispecific BCMAxCD3 T cell engaging antibody, demonstrates robust *in vitro* and *in vivo* anti-tumor efficacy in multiple myeloma models, comparable to that of BCMA CAR T cells. Blood. (2018) 132:1944 10.1182/blood-2018-99-112500

[118] Inc RP First Clinical Data for REGN5458 (BCMAxCD3) Show Positive Preliminary Results in Multiple Myeloma. Available online at: https://www.prnewswire.com/news-releases/first-clinical-data-for-regn5458-bcmaxcd3-show-positive-preliminary-results-in-multiple-myeloma-300971020.html

[119] Klein C Schaefer W Regula JT Dumontet C Brinkmann U Bacac M. Engineering therapeutic bispecific antibodies using CrossMab technology. Methods. (2019) 154:21–31. 10.1016/j.ymeth.2018.11.008 30453028

[120] Costa L First clinical study of the B-cell maturation antigen (BCMA) 2+1 T cell engager (TCE) CC-93269 in patients (Pts) with relapsed/refractory multiple myeloma (RRMM): interim results of a phase 1 multicenter trial. In: ASH. Available online at: https://ash.confex.com/ash/2019/webprogram/Paper122895.html (accessed January 26, 2020).

[121] NCI Drug Dictionary National Cancer Institute (2011) Available online at: https://www.cancer.gov/publications/dictionaries/cancer-drug (accessed July 5, 2019).

[122] Labrijn AF Meesters JI de Goeij BECG van den Bremer ETJ Neijssen J van Kampen MD. Efficient generation of stable bispecific IgG1 by controlled Fab-arm exchange. Proc Natl Acad Sci USA. (2013) 110:5145–50. 10.1073/pnas.1220145110 23479652 PMC3612680

[123] Girgis S Shetty S Jiao T Amuzie C Weinstock D Watson RG Exploratory pharmacokinetic/pharmacodynamic and tolerability study of BCMAxCD3 in cynomolgus monkeys. Blood. (2016) 128:5668 10.1182/blood.V128.22.5668.5668

[124] Topp MS Attal M Langer C Moreau P Facon T Düll J Phase 1 dose-escalation study of BI 836909, an anti-BCMA bi-specific T-cell engager, in relapsed and/or refractory multiple myeloma (RRMM). JCO. (2016) 34:TPS8067 10.1200/JCO.2016.34.15_suppl.TPS8067

[125] Hipp S Tai Y-T Blanset D Deegen P Wahl J Thomas O A novel BCMA/CD3 bispecific T-cell engager for the treatment of multiple myeloma induces selective lysis *in vitro* and *in vivo*. Leukemia. (2017) 31:1743–51. 10.1038/leu.2016.388 28025583

[126] Hipp S Deegen P Wahl J Blanset D Thomas O Rattel B BI 836909, a novel bispecific T cell engager for the treatment of multiple myeloma induces highly specific and efficacious lysis of multiple myeloma cells *in vitro* and shows anti-tumor activity *in vivo*. Blood. (2015) 126:2999 10.1182/blood.V126.23.2999.2999

[127] Topp MS Duell J Zugmaier G Attal M Moreau P Langer C. Anti–B-cell maturation antigen BiTE molecule AMG 420 induces responses in multiple myeloma. J Clin Oncol. (2020) 38:775–783. 10.1200/JCO.19.02657 31895611

[128] Bispecific T-cell Engager AMG 420 Active, Safe, in Heavily Pretreated Multiple Myeloma,. (2019). Available online at: https://www.healio.com/hematology-oncology/myeloma/news/online/{0742fbb9-6a05-4888-adf0-9482e85cbbc2}/bispecific-t-cell-engager-amg-420-active-safe-in-heavily-pretreated-multiple-myeloma (accessed August 19, 2019).

[129] Topp MS Duell J Zugmaier G Attal M Moreau P Langer C Treatment with AMG 420, an anti-B-cell maturation antigen (BCMA) bispecific T-cell engager (BiTE®) antibody construct, induces minimal residual disease (MRD) negative complete responses in relapsed and/or refractory (R/R) multiple myeloma (MM) patients: results of a first-in-human (FIH) phase I dose escalation study. Blood. (2018) 132:1010 10.1182/blood-2018-99-109769

[130] Amgen Oncology | HLE BiTE Available online at: https://www.amgenoncology.com/modalities/hlebite.html (accessed January 17, 2020).

[131] Anti-BCMA BiTE AMG 701 Shows Preclinical Promise in Multiple Myeloma Available online at: https://www.onclive.com/conference-coverage/imw-2019/antibcma-bite-amg-701-shows-preclinical-promise-in-multiple-myeloma (accessed February 14, 2020).

[132] Gantke T Reusch U Ellwanger K Fucek I Weichel M Treder M AFM26 - A novel CD16A-directed bispecific antibody targeting BCMA for multiple myeloma. In: American Association for Cancer Research Annual Meeting. Poster abstract #5671 (2017).

[133] Watkins-Yoon J CTX-8573, an innate-cell engager targeting BCMA, is a highly potent multispecific antibody for the treatment of multiple myeloma. In: ASH. Available online at: https://ash.confex.com/ash/2019/webprogram/Paper128749.html (accessed January 22, 2020).

[134] Kokenyesi R Bernfield M. Core protein structure and sequence determine the site and presence of heparan sulfate and chondroitin sulfate on syndecan-1. J Biol Chem. (1994) 269:12304–9. 8163535

[135] Gharbaran R. Advances in the molecular functions of syndecan-1 (SDC1/CD138) in the pathogenesis of malignancies. Crit Rev Oncol Hematol. (2015) 94:1–17. 10.1016/j.critrevonc.2014.12.003 25563413

[136] Saunders S Jalkanen M O'Farrell S Bernfield M. Molecular cloning of syndecan, an integral membrane proteoglycan. J Cell Biol. (1989) 108:1547–56. 10.1083/jcb.108.4.1547 2494194 PMC2115498

[137] O'Connell FP Pinkus JL Pinkus GS. CD138 (syndecan-1), a plasma cell marker immunohistochemical profile in hematopoietic and nonhematopoietic neoplasms. Am J Clin Pathol. (2004) 121:254–63. 10.1309/617DWB5GNFWXHW4L 14983940

[138] Zhan F Tian E Bumm K Smith R Barlogie B Shaughnessy J. Gene expression profiling of human plasma cell differentiation and classification of multiple myeloma based on similarities to distinct stages of late-stage B-cell development. Blood. (2003) 101:1128–40. 10.1182/blood-2002-06-1737 12393520

[139] Sanderson RD Yang Y. Syndecan-1: a dynamic regulator of the myeloma microenvironment. Clin Exp Metastasis. (2008) 25:149–59. 10.1007/s10585-007-9125-3 18027090 PMC3633534

[140] Jourdan M Ferlin M Legouffe E Horvathova M Liautard J Rossi JF. The myeloma cell antigen syndecan-1 is lost by apoptotic myeloma cells. Br J Haematol. (1998) 100:637–46. 10.1046/j.1365-2141.1998.00623.x 9531328

[141] Beauvais DM Jung O Yang Y Sanderson RD Rapraeger AC. Syndecan-1 (CD138) suppresses apoptosis in multiple myeloma by activating IGF1 receptor: prevention by SynstatinIGF1R inhibits tumor growth. Cancer Res. (2016) 76:4981–93. 10.1158/0008-5472.CAN-16-0232 27364558 PMC5010496

[142] Moreaux J Sprynski A-C Dillon SR Mahtouk K Jourdan M Ythier A. APRIL and TACI interact with syndecan-1 on the surface of multiple myeloma cells to form an essential survival loop. Eur J Haematol. (2009) 83:119–29. 10.1111/j.1600-0609.2009.01262.x 19456850

[143] Seidel C Børset M Hjertner Ø Cao D Abildgaard N Hjorth-Hansen H. High levels of soluble syndecan-1 in myeloma-derived bone marrow: modulation of hepatocyte growth factor activity. Blood. (2000) 96:3139–46. 10.1182/blood.V96.9.3139.h8003139_3139_3146 11049995

[144] Dhodapkar MV Kelly T Theus A Athota AB Barlogie B Sanderson RD. Elevated levels of shed syndecan-1 correlate with tumour mass and decreased matrix metalloproteinase-9 activity in the serum of patients with multiple myeloma. Br J Haematol. (1997) 99:368–71. 10.1046/j.1365-2141.1997.3893203.x 9375756

[145] Lovell R Dunn JA Begum G Barth NJ Plant T Moss PA. Soluble syndecan-1 level at diagnosis is an independent prognostic factor in multiple myeloma and the extent of fall from diagnosis to plateau predicts for overall survival. Br J Haematol. (2005) 130:542–8. 10.1111/j.1365-2141.2005.05647.x 16098068

[146] Dhodapkar KM Krasovsky J Williamson B Dhodapkar MV. Antitumor monoclonal antibodies enhance cross-presentation ofcCellular antigens and the generation of myeloma-specific killer T cells by dendritic cells. J Exp Med. (2002) 195:125–33. 10.1084/jem.20011097 11781371 PMC2196013

[147] Reid S Yang S Brown R Kabani K Aklilu E Ho PJ. Characterisation and relevance of CD138-negative plasma cells in plasma cell myeloma. Int J Lab Hematol. (2010) 32:e190. 10.1111/j.1751-553X.2010.01222.x 20201998

[148] Jagannath S Chanan-Khan A Heffner LT Avigan D Zimmerman TM Lonial S BT062, an antibody-drug conjugate directed against CD138, shows clinical activity in patients with relapsed or relapsed/refractory multiple myeloma. Blood. (2011) 118:305 10.1182/blood.V118.21.305.305

[149] Deaglio S Mehta K Malavasi F. Human CD38: a (r)evolutionary story of enzymes and receptors. Leukemia Res. (2001) 25:1–12. 10.1016/S0145-2126(00)00093-X 11137554

[150] Orciani M Trubiani O Guarnieri S Ferrero E Primio RD. CD38 is constitutively expressed in the nucleus of human hematopoietic cells. J Cell Biochem. (2008) 105:905–12. 10.1002/jcb.21887 18759251

[151] Lee HC. Structure and enzymatic functions of human CD38. Mol Med. (2006) 12:317–23. 10.2119/2006-00086.Lee 17380198 PMC1829193

[152] Deaglio S Morra M Mallone R Ausiello CM Prager E Garbarino G. Human CD38 (ADP-ribosyl cyclase) is a counter-receptor of CD31, an Ig superfamily member. J Immunol. (1998) 160:395–402. 9551996

[153] Dianzani U Funaro A DiFranco D Garbarino G Bragardo M Redoglia V. Interaction between endothelium and CD4+CD45RA+ lymphocytes. Role of the human CD38 molecule. J Immunol. (1994) 153:952–9. 7913116

[154] van de Donk NWCJ Usmani SZ. CD38 antibodies in multiple myeloma: mechanisms of action and modes of resistance. Front Immunol. (2018) 9:2134. 10.3389/fimmu.2018.02134 30294326 PMC6158369

[155] An G Jiang H Acharya C Zhong MY Cai T Yang G SAR 650984, a therapeutic anti-CD38 monoclonal antibody, blocks CD38-CD31 interaction in multiple myeloma. Blood. (2014) 124:4729 10.1182/blood.V124.21.4729.4729

[156] Moretti P Skegro D Ollier R Wassmann P Aebischer C Laurent T BEAT® the bispecific challenge: a novel and efficient platform for the expression of bispecific IgGs. BMC Proc. (2013) 7(Suppl. 6):09 10.1186/1753-6561-7-S6-O9

[157] Richter J Landgren O Auh JK Back J Salhi Y Reddy VA Phase 1, multicenter, open-label study of single-agent bispecific antibody T-cell engager GBR 1342 in relapsed/refractory multiple myeloma. J Clin Oncol. (2018) 36(15 Suppl.). 10.1200/JCO.2018.36.15_suppl.TPS3132

[158] Pharmaceuticals Glenmark Glenmark Pharmaceuticals Announces Decision to Launch Phase 1 Trial in Solid Tumors for its CD38xCD3 Bispecific Antibody GBR 1342 Based on Human Translational Data. Available online at: https://www.prnewswire.com/news-releases/glenmark-pharmaceuticals-announces-decision-to-launch-phase-1-trial-in-solid-tumors-for-its-cd38xcd3-bispecific-antibody-gbr-1342-based-on-human-translational-data-300744934.html (accessed August 15, 2019).

[159] Investigational Antibody GBR 1342 Gets Orphan Drug Designation for MM Myeloma Research News. (2019). Available online at: https://myelomaresearchnews.com/2019/09/20/investigational-antibody-gbr-1342-receives-orphan-drug-designation-for-multiple-myeloma/ (accessed November 28, 2019).

[160] MarketScreener Sorrento Therapeutics : Form of Prospectus Disclosing Information, Facts, Events Covered in Both Forms 424B2, 424B3 | MarketScreener. Available online at: https://www.marketscreener.com/SORRENTO-THERAPEUTICS-INC-14708339/news/Sorrento-Therapeutics-Form-of-prospectus-disclosing-information-facts-events-covered-in-both-for-28828910/ (accessed January 26, 2020).

[161] Tedder TF. CD19: a promising B cell target for rheumatoid arthritis. Nat Rev Rheumatol. (2009) 5:572–7. 10.1038/nrrheum.2009.184 19798033

[162] Wang K Wei G Liu D. CD19: a biomarker for B cell development, lymphoma diagnosis and therapy. Exp Hematol Oncol. (2012) 1:36. 10.1186/2162-3619-1-36 23210908 PMC3520838

[163] Fujimoto M Poe JC Inaoki M Tedder TF. CD19 regulates B lymphocyte responses to transmembrane signals. Semin Immunol. (1998) 10:267–77. 10.1006/smim.1998.9999 9695183

[164] Poe JC Minard-Colin V Kountikov EI Haas KM Tedder TF. A c-Myc and surface CD19 signaling amplification loop promotes B cell lymphoma development and progression in mice. J Immunol. (2012) 189:2318–25. 10.4049/jimmunol.1201000 22826319 PMC3426298

[165] Tedder TF Inaoki M Sato S. The CD19–CD21 complex regulates signal transduction thresholds governing humoral immunity and autoimmunity. Immunity. (1997) 6:107–18. 10.1016/S1074-7613(00)80418-5 9047233

[166] Del Nagro CJ Otero DC Anzelon AN Omori SA Kolla RV Rickert RC. CD19 function in central and peripheral B-cell development. Immunol Res. (2005) 31:119–31. 10.1385/IR:31:2:119 15778510

[167] van Zelm MC Reisli I van der Burg M Castaño D van Noesel CJM van Tol MJD. An antibody-deficiency syndrome due to mutations in the CD19 gene. N Engl J Med. (2006) 354:1901–12. 10.1056/NEJMoa051568 16672701

[168] Haas KM Tedder TF. Role of the CD19 and CD21/35 receptor complex in innate immunity, host defense and autoimmunity. Adv Exp Med Biol. (2005) 560:125–39. 10.1007/0-387-24180-9_16 15934172

[169] Ishiura N Nakashima H Watanabe R Kuwano Y Adachi T Takahashi Y. Differential phosphorylation of functional tyrosines in CD19 modulates B-lymphocyte activation. Eur J Immunol. (2010) 40:1192–204. 10.1002/eji.200939848 20101619

[170] Ishikawa H Tsuyama N Mahmoud MS Fujii R Abroun S Liu S. CD19 expression and growth inhibition of tumours in human multiple myeloma. Leuk Lymphoma. (2002) 43:613–6. 10.1080/10428190290012146 12002767

[171] Garfall AL Maus MV Hwang W-T Lacey SF Mahnke YD Melenhorst JJ. Chimeric antigen receptor T cells against CD19 for multiple myeloma. N Engl J Med. (2015) 373:1040–7. 10.1056/NEJMoa1504542 26352815 PMC4646711

[172] Pratz KW Blinatumomab induced response of multiply refractory multiple myeloma in the context of secondary pre-B cell acute lymphoblastic leukemia. Ann Hematol Oncol. (2017) 4:1174 10.26420/annhematoloncol.2017.1174

[173] Nerreter T Letschert S Götz R Doose S Danhof S Einsele H. Super-resolution microscopy reveals ultra-low CD19 expression on myeloma cells that triggers elimination by CD19 CAR-T. Nat Commun. (2019) 10:3137. 10.1038/s41467-019-10948-w 31316055 PMC6637169

[174] Benjamin JE Stein AS. The role of blinatumomab in patients with relapsed/refractory acute lymphoblastic leukemia. Ther Adv Hematol. (2016) 7:142–56. 10.1177/2040620716640422 27247755 PMC4872177

[175] Research C for DE and FDA Grants Regular Approval to Blinatumomab and Expands Indication to Include Philadelphia Chromosome-Positive B Cell FDA (2018). Available online at: http://www.fda.gov/drugs/resources-information-approved-drugs/fda-grants-regular-approval-blinatumomab-and-expands-indication-include-philadelphia-chromosome (accessed August 24, 2019).

[176] Kantarjian H Stein A Gökbuget N Fielding AK Schuh AC Ribera J-M. Blinatumomab versus chemotherapy for advanced acute lymphoblastic leukemia. N Engl J Med. (2017) 376:836–47. 10.1056/NEJMoa1609783 28249141 PMC5881572

[177] Intervention Dynamic Trial Listing Page National Cancer Institute (2017). Available online at: https://www.cancer.gov/about-cancer/treatment/clinical-trials/intervention/blinatumomab (accessed August 24, 2019).

[178] Kumaresan PR Lai WC Chuang SS Bennett M Mathew PA. CS1, a novel member of the CD2 family, is homophilic and regulates NK cell function. Mol Immunol. (2002) 39:1–8. 10.1016/S0161-5890(02)00094-9 12213321

[179] Lee JK Mathew SO Vaidya SV Kumaresan PR Mathew PA. CS1 (CRACC, CD319) induces proliferation and autocrine cytokine expression on human B lymphocytes. J Immunol. (2007) 179:4672–8. 10.4049/jimmunol.179.7.4672 17878365

[180] Veillette A SLAM-family receptors: immune regulators with or without SAP-family adaptors. Cold Spring Harb Perspect Biol. (2010) 2:a002469 10.1101/cshperspect.a002469 20300214 PMC2829957

[181] De Salort J Sintes J Llinàs L Matesanz-Isabel J Engel P. Expression of SLAM (CD150) cell-surface receptors on human B-cell subsets: from pro-B to plasma cells. Immunol Lett. (2011) 134:129–36. 10.1016/j.imlet.2010.09.021 20933013

[182] Hsi ED Steinle R Balasa B Szmania S Draksharapu A Shum BP. CS1, a potential new therapeutic antibody target for the treatment of multiple myeloma. Clin Cancer Res. (2008) 14:2775–84. 10.1158/1078-0432.CCR-07-4246 18451245 PMC4433038

[183] Tai Y-T Dillon M Song W Leiba M Li X-F Burger P. Anti-CS1 humanized monoclonal antibody HuLuc63 inhibits myeloma cell adhesion and induces antibody-dependent cellular cytotoxicity in the bone marrow milieu. Blood. (2008) 112:1329–37. 10.1182/blood-2007-08-107292 17906076 PMC2515112

[184] Szmania S Balasa B Malaviarachchi P Zhan F Huang Y Draksharapu A CS1 is expressed on myeloma cells from early stage, late stage, and drug-treated multiple myeloma patients, and is selectively targeted by the HuLuc63 antibody. Blood. (2006) 108:660 10.1182/blood.V108.11.660.660

[185] Veillette A Guo H. CS1, a SLAM family receptor involved in immune regulation, is a therapeutic target in multiple myeloma. Crit Rev Oncol Hematol. (2013) 88:168–77. 10.1016/j.critrevonc.2013.04.003 23731618

[186] López-Larrea C Suárez-Alvarez B López-Soto A López-Vázquez A Gonzalez S. The NKG2D receptor: sensing stressed cells. Trends Mol Med. (2008) 14:179–89. 10.1016/j.molmed.2008.02.004 18353724

[187] Franco A Damdinsuren B Ise T Dement-Brown J Li H Nagata S. Human Fc receptor-like 5 binds intact IgG via mechanisms distinct from that of Fc-receptors. J Immunol. (2013) 190:5739–46. 10.4049/jimmunol.1202860 23616577 PMC3660407

[188] Li J Stagg NJ Johnston J Harris MJ Menzies SA DiCara D. Membrane-proximal epitope facilitates efficient T cell synapse formation by anti-FcRH5/CD3 and is a requirement for myeloma cell killing. Cancer Cell. (2017) 31:383–95. 10.1016/j.ccell.2017.02.001 28262555 PMC5357723

[189] Sawyer JR Tricot G Mattox S Jagannath S Barlogie B. Jumping translocations of chromosome 1q in multiple myeloma: evidence for a mechanism involving decondensation of pericentromeric heterochromatin. Blood. (1998) 91:1732–41. 10.1182/blood.V91.5.1732.1732_1732_1741 9473240

[190] Hatzivassiliou G Miller I Takizawa J Palanisamy N Rao PH Iida S. IRTA1 and IRTA2, novel immunoglobulin superfamily receptors expressed in B cells and involved in chromosome 1q21 abnormalities in B cell malignancy. Immunity. (2001) 14:277–89. 10.1016/S1074-7613(01)00109-1 11290337

[191] Bluemel C Hausmann S Fluhr P Sriskandarajah M Stallcup WB Baeuerle PA. Epitope distance to the target cell membrane and antigen size determine the potency of T cell-mediated lysis by BiTE antibodies specific for a large melanoma surface antigen. Cancer Immunol Immunother. (2010) 59:1197–209. 10.1007/s00262-010-0844-y 20309546 PMC11030089

[192] Sun LL Ellerman D Mathieu M Hristopoulos M Chen X Li Y. Anti-CD20/CD3 T cell–dependent bispecific antibody for the treatment of B cell malignancies. Sci Transl Med. (2015) 7:287ra70. 10.1126/scitranslmed.aaa4802 25972002

[193] Ovacik AM Li J Lemper M Danilenko D Stagg N Mathieu M. Single cell-produced and *in vitro*-assembled anti-FcRH5/CD3 T-cell dependent bispecific antibodies have similar *in vitro* and *in vivo* properties. MAbs. (2019) 11:422–33. 10.1080/19420862.2018.1551676 30550367 PMC6380433

[194] Atamaniuk J Gleiss A Porpaczy E Kainz B Grunt TW Raderer M. Overexpression of G protein-coupled receptor 5D in the bone marrow is associated with poor prognosis in patients with multiple myeloma. Eur J Clin Invest. (2012) 42:953–60. 10.1111/j.1365-2362.2012.02679.x 22591013

[195] NCI Thesaurus Available online at: https://ncithesaurus.nci.nih.gov/ncitbrowser/ConceptReport.jsp?dictionary=NCI_Thesaurus&version=19.10d&ns=ncit&code=C147936&key=124273150&b=1&n=null (accessed November 29, 2019).

[196] Bräuner-Osborne H Jensen AA Sheppard PO Brodin B Krogsgaard-Larsen P O'Hara P. Cloning and characterization of a human orphan family C G-protein coupled receptor GPRC5D. Biochim Biophys Acta. (2001) 1518:237–48. 10.1016/S0167-4781(01)00197-X 11311935

[197] NCI Thesaurus Available online at: https://ncithesaurus.nci.nih.gov/ncitbrowser/pages/message.jsf (accessed November 29, 2019).

[198] Genmab - JNJ-64407564 Available online at: http://www.genmab.com/product-pipeline/products-in-development/jnj-64407564 (accessed November 29, 2019).

[199] Technology Available online at: http://www.genmab.com/duobody/technology#tab1 (accessed November 29, 2019).

[200] Pillarisetti K Edavettal S Mendonça M Li Y Tornetta MA Babich A. A T cell redirecting bispecific g-protein coupled receptor class 5 member DxCD3 antibody to treat multiple myeloma. Blood. (2020) 135:1232–43. 10.1182/blood.2019003342. 32040549 PMC7146017

[201] Lauring J Abukhdeir AM Konishi H Garay JP Gustin JP Wang Q. The multiple myeloma–associated MMSET gene contributes to cellular adhesion, clonogenic growth, and tumorigenicity. Blood. (2008) 111:856–64. 10.1182/blood-2007-05-088674 17942756 PMC2200833

[202] Gnjatic S Nishikawa H Jungbluth AA Güre AO Ritter G Jäger E. NY-ESO-1: review of an immunogenic tumor antigen. Adv Cancer Res. (2006) 95:1–30. 10.1016/S0065-230X(06)95001-5 16860654

[203] Thomas R Al-Khadairi G Roelands J Hendrickx W Dermime S Bedognetti D. NY-ESO-1 based immunotherapy of cancer: current perspectives. Front Immunol. (2018) 9:947. 10.3389/fimmu.2018.00947 29770138 PMC5941317

[204] Kisseleva-Romanova E Lopreiato R Baudin-Baillieu A Rousselle J-C Ilan L Hofmann K. Yeast homolog of a cancer-testis antigen defines a new transcription complex. EMBO J. (2006) 25:3576–85. 10.1038/sj.emboj.7601235 16874308 PMC1538566

[205] Cho HJ Caballero OL Gnjatic S Andrade VCC Colleoni GW Vettore AL. Physical interaction of two cancer-testis antigens, MAGE-C1 (CT7) and NY-ESO-1 (CT6). Cancer Immun. (2006) 6:12. 17137291

[206] Barker PA Salehi A. The MAGE proteins: emerging roles in cell cycle progression, apoptosis, and neurogenetic disease. J Neurosci Res. (2002) 67:705–12. 10.1002/jnr.10160 11891783

[207] Cronwright G Le Blanc K Götherström C Darcy P Ehnman M Brodin B. Cancer/testis antigen expression in human mesenchymal stem cells: down-regulation of SSX impairs cell migration and matrix metalloproteinase 2 expression. Cancer Res. (2005) 65:2207–15. 10.1158/0008-5472.CAN-04-1882 15781633

[208] van Rhee F Szmania SM Zhan F Gupta SK Pomtree M Lin P. NY-ESO-1 is highly expressed in poor-prognosis multiple myeloma and induces spontaneous humoral and cellular immune responses. Blood. (2005) 105:3939–44. 10.1182/blood-2004-09-3707 15671442 PMC1895070

[209] Jäger E Chen YT Drijfhout JW Karbach J Ringhoffer M Jäger D. Simultaneous humoral and cellular immune response against cancer-testis antigen NY-ESO-1: definition of human histocompatibility leukocyte antigen (HLA)-A2-binding peptide epitopes. J Exp Med. (1998) 187:265–70. 10.1084/jem.187.2.265 9432985 PMC2212106

[210] Luetkens T Kobold S Cao Y Ristic M Schilling G Tams S. Functional autoantibodies against SSX-2 and NY-ESO-1 in multiple myeloma patients after allogeneic stem cell transplantation. Cancer Immunol Immunother. (2014) 63:1151–62. 10.1007/s00262-014-1588-x 25078248 PMC11029676

[211] Crucian BE Moscinski LC Androlewicz M Ballester OF Widen RH Yu H. Assessment of intracellular TAP-1 and TAP-2 in conjunction with surface MHC class I in plasma cells from patients with multiple myeloma. Br J Haematol. (1997) 98:426–32. 10.1046/j.1365-2141.1997.2173032.x 9266943

[212] Gabathuler R Reid G Kolaitis G Driscoll J Jefferies WA. Comparison of cell lines deficient in antigen presentation reveals a functional role for TAP-1 alone in antigen processing. J Exp Med. (1994) 180:1415–25. 10.1084/jem.180.4.1415 7931074 PMC2191686

[213] Hokland M Basse P Justesen J Hokland P. IFN-induced modulation of histocompatibility antigens on human cells. Background, mechanisms and perspectives. Cancer Metastasis Rev. (1988) 7:193–207. 10.1007/BF00047751 2465842

[214] Routes JM. IFN increases class I MHC antigen expression on adenovirus-infected human cells without inducing resistance to natural killer cell killing. J Immunol. (1992) 149:2372–7. 1382100

[215] Liddy N Bossi G Adams KJ Lissina A Mahon TM Hassan NJ. Monoclonal TCR-redirected tumor cell killing. Nat Med. (2012) 18:980–7. 10.1038/nm.2764 22561687

[216] De Geer A Carlson L-M Kogner P Levitskaya J. Soluble factors released by activated cytotoxic T lymphocytes interfere with death receptor pathways in neuroblastoma. Cancer Immunol Immunother. (2008) 57:731–43. 10.1007/s00262-007-0412-2 17962944 PMC11031004

[217] Martinelli G Boissel N Chevallier P Ottmann O Gökbuget N Topp MS. Complete hematologic and molecular response in adult patients with relapsed/refractory philadelphia chromosome–positive B-precursor acute lymphoblastic leukemia following treatment with blinatumomab: results from a phase II, single-arm, multicenter study. JCO. (2017) 35:1795–802. 10.1200/JCO.2016.69.3531 28355115

[218] Brinkman IH van de Laar MAFJ Jansen TL van Roon EN. The potential risk of infections during (prolonged) rituximab therapy in rheumatoid arthritis. Expert Opin Drug Saf . (2011) 10:715–26. 10.1517/14740338.2011.562188 21401437

[219] Chimeric, Antigen Receptor (CAR) T-Cell Therapy | Leukemia and Lymphoma Society,. Available online at: https://www.lls.org/treatment/types-of-treatment/immunotherapy/chimeric-antigen-receptor-car-t-cell-therapy (accessed November 29, 2019).

[220] Wu L Seung E Xu L Rao E Lord DM Wei RR Trispecific antibodies enhance the therapeutic efficacy of tumor-directed T cells through T cell receptor co-stimulation. Nat Cancer. (2019) 1:86–98. 10.1038/s43018-019-0004-z 35121834

[221] Esensten JH Helou YA Chopra G Weiss A Bluestone JA. CD28 costimulation: from mechanism to therapy. Immunity. (2016) 44:973–88. 10.1016/j.immuni.2016.04.020 27192564 PMC4932896

[222] Hui E Cheung J Zhu J Su X Taylor MJ Wallweber HA. T cell costimulatory receptor CD28 is a primary target for PD-1-mediated inhibition. Science. (2017) 355:1428–33. 10.1126/science.aaf1292 28280247 PMC6286077

[223] Suen H Brown R Yang S Weatherburn C Ho PJ Woodland N. Multiple myeloma causes clonal T-cell immunosenescence: identification of potential novel targets for promoting tumour immunity and implications for checkpoint blockade. Leukemia. (2016) 30:1716–24. 10.1038/leu.2016.84 27102208

[224] Köhnke T Krupka C Tischer J Knösel T Subklewe M. Increase of PD-L1 expressing B-precursor ALL cells in a patient resistant to the CD19/CD3-bispecific T cell engager antibody blinatumomab. J Hematol Oncol. (2015) 8:111. 10.1186/s13045-015-0213-6 26449653 PMC4599591

[225] Görgün G Samur MK Cowens KB Paula S Bianchi G Anderson JE. Lenalidomide enhances immune checkpoint blockade-induced immune response in multiple myeloma. Clin Cancer Res. (2015) 21:4607–18. 10.1158/1078-0432.CCR-15-0200 25979485 PMC4609232

[226] Krupka C Kufer P Kischel R Zugmaier G Lichtenegger FS Köhnke T. Blockade of the PD-1/PD-L1 axis augments lysis of AML cells by the CD33/CD3 BiTE antibody construct AMG 330: reversing a T-cell-induced immune escape mechanism. Leukemia. (2016) 30:484–91. 10.1038/leu.2015.214 26239198

[227] Laszlo GS Gudgeon CJ Harrington KH Walter RB. T-cell ligands modulate the cytolytic activity of the CD33/CD3 BiTE antibody construct, AMG 330. Blood Cancer J. (2015) 5:e340. 10.1038/bcj.2015.68 26295610 PMC4558592

[228] Duell J Dittrich M Bedke T Mueller T Eisele F Rosenwald A. Frequency of regulatory T cells determines the outcome of the T-cell-engaging antibody blinatumomab in patients with B-precursor ALL. Leukemia. (2017) 31:2181–90. 10.1038/leu.2017.41 28119525 PMC5629361

[229] Vallera DA Felices M McElmurry R McCullar V Zhou X Schmohl JU. IL15 trispecific killer engagers (TriKE) make natural killer cells specific to CD33+ targets while also inducing persistence, *in vivo* expansion, and enhanced function. Clin Cancer Res. (2016) 22:3440–50. 10.1158/1078-0432.CCR-15-2710 26847056 PMC4947440

[230] Lum LG Thakur A Kondadasula SV Al-Kadhimi Z Deol A Tomaszewski EN. Targeting CD138–/CD20+ clonogenic myeloma precursor cells decreases these cells and induces transferable antimyeloma immunity. Biol Blood Marrow Transplant. (2016) 22:869–78. 10.1016/j.bbmt.2015.12.030 26827660 PMC6820521

[231] Spisek R Kukreja A Chen L-C Matthews P Mazumder A Vesole D. Frequent and specific immunity to the embryonal stem cell–associated antigen SOX2 in patients with monoclonal gammopathy. J Exp Med. (2007) 204:831–40. 10.1084/jem.20062387 17389240 PMC2118551

[232] Rossi EA Rossi DL Stein R Goldenberg DM Chang C-H. A bispecific antibody-IFNα2b immunocytokine targeting CD20 and HLA-DR is highly toxic to human lymphoma and multiple myeloma cells. Cancer Res. (2010) 70:7600–9. 10.1158/0008-5472.CAN-10-2126 20876805

